# A New Relatively Simple Approach to Multipole Interactions in Either Spherical Harmonics or Cartesians, Suitable for Implementation into Ewald Sums

**DOI:** 10.3390/ijms21010277

**Published:** 2019-12-31

**Authors:** Christian J. Burnham, Niall J. English

**Affiliations:** School of Chemical and Bioprocess Engineering, University College Dublin, Belfield, Dublin 4, Ireland; christian.burnham@ucd.ie

**Keywords:** multipoles, Ewald, molecular simulation, spherical harmonics

## Abstract

We present a novel derivation of the multipole interaction (energies, forces and fields) in spherical harmonics, which results in an expression that is able to exactly reproduce the results of earlier Cartesian formulations. Our method follows the derivations of Smith (W. Smith, *CCP5 Newsletter* 1998, *46*, 18.) and Lin (D. Lin, *J. Chem. Phys.* 2015, *143*, 114115), who evaluate the Ewald sum for multipoles in Cartesian form, and then shows how the resulting expressions can be converted into spherical harmonics, where the conversion is performed by establishing a relation between an inner product on the space of symmetric traceless Cartesian tensors, and an inner product on the space of harmonic polynomials on the unit sphere. We also introduce a diagrammatic method for keeping track of the terms in the multipole interaction expression, such that the total electrostatic energy can be viewed as a ‘sum over diagrams’, and where the conversion to spherical harmonics is represented by ‘braiding’ subsets of Cartesian components together. For multipoles of maximum rank n, our algorithm is found to have scaling of $n^{3.7}$ vs. $n^{4.5}$ for our most optimised Cartesian implementation.

## 1. Introduction

Point-charge electrostatic models have been a mainstay of molecular simulation for years. Increasing in sophistication, many authors have also implemented dipole interactions, such that the electrostatics on each nuclear site is modelled by the combination of a dipole and a charge, which requires that the simulation code be capable of calculating both charge–dipole and dipole–dipole interactions in addition to the usual charge–charge interactions required for point charge models. This presented particular problems for treatment of long-range interactions, as typically handled by an Ewald sum, or related schemes, as such algorithms were initially developed for the case of point charge models, and thus needed to be modified in order to account for dipole interactions. Fortunately, the problem of adapting Ewald sums for dipoles has largely been solved, and, Smith [1] in particular has provided a formulation which is well organised and easy to follow.

In recent years, some groups have moved even beyond the dipole level, exploring the use of yet higher terms in the multipole expansion of the charge distribution, where the terms in the next three ranks are referred to by names quadrupole, octopole and hexadecapole. Again though, the problem is faced—how to implement these terms in a simulation code, and how to modify an Ewald sum, or similar algorithm, such that it can properly handle the higher order multipoles interactions.

There is currently a strong need for such methods, given that there now exist reliable convenient methods to partition the charge distribution from electronic-structure calculations into atomic multipoles residing on each nuclear site, with the most notable method being the Distributed Multipole Analysis (DMA) approach of Stone and coworkers [2,3,4]. DMA provides a particularly appealing route for the construction of accurate empirical models, because researchers should now be able take the values of atomic multipoles as output from a DMA analysis as input for their models. However, doing this depends, of course, on the ability to write code which can calculate the required multipole interactions, and this is far from trivial.

Progress has been made, with popular molecular-dynamics packages, such as DL-POLY [5] and TINKER [6] having functionality to evaluate potential-energy surfaces with multipole interactions. So, in a sense, this is a solved problem, but it is still worth exploring, given the complexity of the current approaches, if there are any ways to simplify the mathematical and computational approaches for computing multipole interactions.

Smith’s method can, in principle, be used up to arbitrary rank, and his original paper even includes the terms up to quadrupole. However, there is a noticeable increase in complexity and computational cost incurred by going up to higher ranks, such that the quadrupole terms are both more difficult to both derive and to calculate than the dipole terms, and, it seems like it would be a significant undertaking to extend this approach to yet higher ranks.

However, it was recently shown by Lin [7] that we do not have to derive the higher multipole terms “by hand”, as it were. Instead, it is possible to derive a more general formula which more or less automatically generates the expression for any degree multipole, without resorting to increasingly tedious algebra.

It is true that past formulations by Smith and others [8,9] are extendable to arbitrarily higher rank multipoles, but Lin’s work is, to our best knowledge, the first to give such a direct closed form expression for the interaction between multipoles of any rank. However, even Lin’s extension to Smith’s approach suffers from a drawback, which is that they both choose to evaluate the multipole interaction in Cartesians. Furthermore, this is a problem because, as it turns out, the Cartesian representation of multipoles is highly redundant. This is due to the presence of symmetries in the Cartesian representation, resulting in the use of far more numbers than is strictly required to represent each multipole, making it hard to calculate the multipole interactions in the minimal number of operations.

Bearing this in mind, it is possible to make many optimisations in the Cartesian implementation, such that these redundancies have relatively minimal impact. Most notably, it is possible to take advantage of the fact that the traces of the Cartesian tensors do not contribute to the total energies/forces of the multipole interaction, and so remove them, an approach that has been used with success by both Lin and Huang [10,11]. It is also possible to write the required sums in such a way that the redundancy from the remaining tensor symmetries is reduced.

However, to increase computational efficiency even further, what is really needed is an expression in which the multipoles are described by a completely non redundant basis. Furthermore, it has long been known that this is provided by moving from a Cartesian representation to spherical harmonics.

It could be argued that a Cartesian-multipole representation is more compatible with existing molecular dynamics codes, given they are normally mostly written in Cartesians. Furthermore, it is certainly true that most people prefer thinking in terms of Cartesians, than the somewhat complex spherical harmonics. However, the spherical harmonics approach ultimately proves to be faster, and more elegant, and so is arguably worth the extra effort for codes which make heavy use of multipoles.

Writing the interaction in terms of spherical harmonics is usually regarded as a formidable task, requiring mastery of the mathematics of manipulating these objects, and intimate knowledge of the spherical harmonics’ properties. Furthermore, the complexity is further multiplied when it comes to modifying the treatment of long-range interactions, such as when handled by Ewald sums. Even so, a derivation of the Ewald sum for multipoles has been given by Leslie [12], who described his implementation of multipoles in the DL-MULTI software package, which can be interfaced with DL-POLY [5] molecular dynamics software package. Leslie gave the expression for the Ewald sum form of the multipole interaction in terms of Stone’s S functions. These functions, which are derived in ref. [13], act give the interaction tensor components between two multipoles on different sites, each described in their local reference frames, where the necessary orientational information is described by Wigner rotation matrices, and where the S function also contains a spherical harmonic, which acts to mediate the interaction between.

Another derivation has recently been given by Simmonett et al. [14] who proposed a method for converting the Ewald sum into the spherical harmonic formulation of Stone, which required the aid of symbolic algebra package to derive. Furthermore, the resulting expression requires the calculation of hard to interpret ‘contamination terms’ in the tensorial interactions between the multipoles, which arise from the modification of the multipole interaction due to the Gaussian screening functions that are present in the real-space part of the Ewald sum. Although the above derivations appear complete and satisfactory, they do depend on fairly technical knowledge, which is presumably why many in the community have been slow to implement such approaches.

In the present contribution, we aim to rectify this situation by providing a surprisingly straightforward derivation of the multipole interaction in spherical harmonics which, unusually, does not require any detailed technical knowledge of the theory of spherical harmonics. Our method provides a direct connection between the Cartesian and spherical harmonic representations, such that it becomes straightforward to transform between the two representations, and our final expressions lend themselves to being easily implemented in Ewald sum type methods. Now, we seek to provide a comprehensive account of the full development of this Cartesian formulation, so this will necessarily involve some presentation of relatively well-known, pervious material in the theory and (spherical-harmonics) presentation and treatment of multipoles. Still, in the present contribution, we wish to provide a full, and self-contained development of a Cartesian formulation, in addition to an intuitive associated graphical representation.

We do not claim that the resulting expressions are superior to previous derivations, such as that given by Leslie and Simmonett et al.; we are full of admiration for their work. However, the approach presented here is significantly different to standard derivations, which makes it interesting in itself, and our resulting expressions are also in a different form, though they must be completely equivalent in their predictions, to those obtained with other methods. We also think our approach will be of interest in showing how the Smith and Lin method for Cartesian multipoles can be transformed into a spherical harmonic equivalent, while preserving the essential structure of their solution.

It would, of course, be of enormous interest to find a reasonably direct way of connecting our expressions for the multipole interaction in spherical harmonics to those produced by other derivations. However, this appears far from trivial, given we have taken what seems to be such a radically different approach to that used by other authors.

Our approach is also very much inspired by the work of Applequist [15,16], who published two papers detailing some quite beautiful theorems on the deep connection between homogeneous polynomials and Cartesian tensors, and also on the properties of the Maxwell Cartesian spherical harmonics, which are the natural Cartesian analogues of the spherical harmonics, both of which we will have cause to use in the course of this work.

Our approach is summarised as follows. We first essentially follow the approaches of Smith and Lin to give the multipole interactions in terms of Cartesians. We then proceed to show how this interaction can be converted into spherical harmonic form. This is done with the aid of three key ideas. (i) the use of a diagrammatic representation of the interaction between multipole sites, which greatly clarifies the math, as it turns out the complete interaction can be calculated in a ‘sum over diagrams’ type sense. (ii) the use of spherical harmonics to construct an orthogonal basis for the traceless multipole tensors, such that the Cartesian to spherical harmonic conversion can be achieved by way of Stone’s tables of spherical harmonics, as given in reference [13]. (iii) the recognition that the multipole interaction can be written in terms of so-called tensor inner products, which we will show are proportional to inner products involving spherical harmonics, allowing us to convert the entire multipole interaction into spherical-harmonic form.

## 2. Results

### 2.1. The Multipole Expansion

Consider a cluster of $N_{c}$ charges, with charges $q_{m}$, at positions $r+\Delta r_{m}$, where the Δ is used to signify that the displacements are typically quite small (at least, they are closer to r than any test sites probing the fields from the distribution). We then define the rank-n Cartesian multipole tensors (*n* = 0,1,…), $M^{\left(n\right)}$, of the distribution, with respect to r, according to

(1) $$M^{\left(n\right)}=\frac{1}{n!}\overset{N_{c}}{\underset{m=1}{\sum^{}}}q_{m}\Delta R^{m\left(n\right)}$$

where $\Delta R^{m\left(n\right)}=\Delta r^{m}\Delta r^{m}\Delta r^{m}..$ is a tensor product, with Cartesian components $\Delta R_{\alpha \beta \gamma ..}^{m\left(n\right)}=\Delta r_{\alpha }^{m}\Delta r_{\beta }^{m}\Delta r_{\gamma }^{m}..$, in which each Greek index is one of x,y,z, and there being n factors of type $\Delta r_{\alpha }^{m}$ in the product. Furthermore, where, for *n* = 0, the tensor becomes unity: $\Delta R^{m\left(0\right)}=1$ (see Section 4.1).

Note: In Equation (1), and throughout, we are following Smith in subsuming the inverse factorial in the definition of each multipole, but we warn that other authors use different conventions, and it is important to check which convention is being used when comparing different works.

The rank 0 multipole, $M^{\left(0\right)}$, which is a scalar, is just the sum of the charge. The rank 1 multipole $M^{\left(1\right)}$ multipole is referred to as the *dipole* moment, and is a vector with components $M_{x}^{\left(1\right)}$, $M_{y}^{\left(1\right)}$, $M_{z}^{\left(1\right)}$. The rank 2 multipole, $M^{\left(2\right)}$ is called the *quadrupole*, and has 9 components: $M_{xx}^{\left(2\right)}$, $M_{xy}^{\left(2\right)}$, $M_{xz}^{\left(2\right)}$, etc., and the rank 3 multipole with 27 components is called the *hexadecapole*, and so on.

It is clear from their definition in Equation (1). that each multipole tensor is symmetric under any permutation of its indices; e.g., for the rank-3 multipole tensor, $M_{\alpha \beta \gamma }^{\left(3\right)}=M_{\alpha \gamma \beta }^{\left(3\right)}$ $=M_{\beta \gamma \alpha }^{\left(3\right)}=M_{\beta \alpha \gamma }^{\left(3\right)}$ $=M_{\gamma \alpha \beta }^{\left(3\right)}=M_{\gamma \beta \alpha }^{\left(3\right)}$. Furthermore, it can be shown that the same is true in any axis frame. This permutation symmetry means that the multipole tensors belong to a class that are referred to as *symmetric* tensors, and we will have frequent cause to make use of this symmetry throughout this work.

The multipoles are useful because the electrostatic properties of the cluster can often be written in terms of a rapidly converging series over multipole moments of increasing rank, rather than having to sum over the individual charges themselves. To see this, let us place the charge distribution in a background non-uniform electrostatic potential, ϕ(r). Then, the electrostatic energy, U(r), of the cluster due to ϕ(r) is given by

(2) $$U\left(r\right)=\underset{m}{\sum^{}}q_{m}\phi \left(r+\Delta r_{m}\right)$$

Now, using the inner product notation $\langle A^{\left(n\right)},B^{\left(n\right)}\rangle_{t}$, where $\langle A^{\left(n\right)},B^{\left(n\right)}\rangle_{t}$ indicates an inner product over tensors $A^{\left(n\right)}$ and $B^{\left(n\right)}$ (see Section 4.1)

(3) $$\langle A^{\left(n\right)},B^{\left(n\right)}\rangle_{t}=\underset{\alpha \beta \gamma ..}{\sum^{}}A_{\alpha \beta \gamma ..}^{\left(n\right)}B_{\alpha \beta \gamma ..}^{\left(n\right)}$$

the Taylor series expansion of $\phi \left(r+\Delta r_{m}\right)$ can be written as

(4) $$\phi \left(r+\Delta r\right)=\overset{\infty }{\underset{n=0}{\sum^{}}}\frac{1}{n!}\langle \Delta R^{\left(n\right)},\nabla^{\left(n\right)}\rangle_{t}\phi \left(r\right)$$

where $\nabla^{\left(n\right)}=\nabla \nabla \nabla ..$ with components $\nabla_{\alpha \beta \gamma ..}^{\left(n\right)}=\partial^{n}/\partial r_{\alpha }\partial r_{\beta }\partial r_{\gamma }..$ (again, see Section 4.1).

Now, defining the rank n symmetric electrostatic field tensors $\phi^{\left(n\right)}\left(r\right)$ from

(5) $$\phi^{\left(n\right)}\left(r\right)=\nabla^{\left(n\right)}\phi \left(r\right)$$

Furthermore, substituting Equation (4) into Equation (2) and using Equation (1), we can rewrite the energy expression as

(6) $$U\left(r\right)=\overset{\infty }{\underset{n=0}{\sum^{}}}\langle M^{\left(n\right)},\phi^{\left(n\right)}\left(r\right)\rangle_{t},$$

Similarly, it can be shown from the elementary theory of multipoles, and from using another Taylor series expansion, that the electrostatic potential at r, from a charge cluster around the origin, with multipoles $M^{\left(n\right)}$ is given by

(7) $$\phi \left(r\right)=\overset{\infty }{\underset{n=0}{\sum^{}}}{\left(-1\right)}^{n}\langle M^{\left(n\right)},\nabla^{\left(n\right)}\rangle_{t}\left\{\frac{1}{r}\right\}.$$

where we have set $4\pi \epsilon_{0}=1$ to simplify the formulae.

Thus, assuming the above series converges, and we wish to calculate the electrostatic potential from the charge cluster, we are justified in ignoring the fine details of the charge distribution and just working with its multipoles up to a maximum rank, where these multipoles are calculated from the multipole expansion of the charge distribution in Equation (1), and where we imagine the multipoles are placed on the multipole expansion site, in this case at the origin.

### 2.2. Traceless Tensors and the Detracing Operator

The trace of a rank 2 tensor is given by the sum of its diagonal elements,

(8) $$Tr\left(A^{\left(2\right)}\right)=\underset{\alpha }{\sum^{}}A_{\alpha \alpha }^{\left(2\right)}.$$

In general, and following Applequist [15] (Section 2.3), we will define the trace of a rank *n* symmetric Cartesian tensor as

(9) $$Tr{\left(A^{\left(n\right)}\right)}_{\alpha \beta \gamma ..}=\underset{\chi }{\sum^{}}A_{\alpha \beta \gamma ..\chi \chi }^{\left(n\right)},$$

where the trace tensor, $Tr\left(A^{\left(n\right)}\right)$, is a symmetric tensor of rank *n* − 2.

Tensors for which $Tr\left(A^{\left(n\right)}\right)$ = 0 will be referred to as *traceless*. In fact, we have already encountered two such tensors: (i) the $\phi^{\left(n\right)}\left(r\right)$ tensor, defined in Equation (5) above, and (ii) the $\nabla^{\left(n\right)}\left\{1/r\right\}$ tensor which occurs in Equation (7).

To show that the $\phi^{\left(n\right)}\left(r\right)$ tensor is traceless, we combine Equations (5) and (9) to obtain

(10) $$Tr{\left(\phi^{\left(n\right)}\right)}_{\alpha \beta \gamma ..}=\nabla_{\alpha \beta \gamma ..}^{\left(n-2\right)}\nabla^{2}\phi \left(r\right)=0,$$

where we have used the fact that the electrostatic potential in free space is a solution to Laplace’s eqn, $\nabla^{2}\phi \left(r\right)=0$. Thus, $\phi^{\left(n\right)}\left(r\right)$ is traceless, and, given that 1/r is just the potential from a unit charge, a similar argument shows that $\nabla^{\left(n\right)}\left\{1/r\right\}$ is also traceless.

As an aside, it may be argued that in real systems the Laplacian is not zero, because any real atomic site will experience a non-zero charge-density from the inter-atomic and inter-molecular charge-clouds from the other electrons. However, the standard multipole expansion for the interatomic electrostatic energy does not account for such effects, and they will henceforth be assumed to be zero.

The addition of any two rank n traceless tensors, or the multiplication of a traceless tensor by a scalar will result in another traceless tensor. Thus, the traceless tensors of rank n form a *vector subspace* of rank n tensors. As such we ought to be able to find a projection operator which projects out the non-traceless part of any rank n symmetric tensor. For example, we might (correctly) guess that the rank 2 tensor $A^{\left(2\right)}$ has the traceless projection $A_{\alpha \beta }^{t\left(2\right)}=r_{\alpha }r_{\beta }-Tr\left\{A^{\left(2\right)}\right\}\delta_{\alpha ,\beta }/3$. Furthermore, we will now show how to find this projection in general.

Before we begin, it is convenient to introduce $R^{\left(n\right)}$ tensors, $R^{\left(n\right)}=rrrr..$, with components $R_{\alpha \beta \gamma ..}^{\left(n\right)}=r_{\alpha }r_{\beta }r_{\gamma }r_{\delta }..$, and where $R^{\left(0\right)}=1$ by convention (see Section 4.1).

We will now make use of a theorem due to Hobson, [17] which has been extensively discussed by Applequist, ref [15,16] that the projection of the $R^{\left(n\right)}$ tensors onto the subspace of traceless tensors is given by

(11) $$R^{t\left(n\right)}=\hat{D}R^{\left(n\right)}={\left(-1\right)}^{n}\frac{r^{2n+1}}{\left(2n-1\right)!!}\nabla^{\left(n\right)}\left\{\frac{1}{r}\right\}$$

where $\hat{D}$ is the so-called *detracing* projection operator onto the subspace of traceless tensors, and where $R^{t\left(n\right)}=\hat{D}R^{\left(n\right)}$ is the traceless tensor resulting from the projection of $R^{\left(n\right)}$ into that traceless subspace. Furthermore, where that $R^{t\left(n\right)}$ is traceless follows immediately from the traceless nature of $\nabla^{\left(n\right)}\left\{1/r\right\}$.

Applequist refers to $R^{t\left(n\right)}$ as the *Maxwell Cartesian spherical harmonics*, given that these gradients were investigated by James Clerk Maxwell [18], and given that, although they are not orthogonal, they behave in many senses like the Cartesian analogue of the spherical harmonic polynomials, of which more later.

For the moment we can think of the $\hat{D}$ operator as being defined by Equation (11), and so our job is to show that $\hat{D}$ defined this way is indeed the detracing projection operator.

Firstly, we need a way to express the action of $\hat{D}$ on a general degree n tensor. Applequist has provided a closed-form expression for the matrix representation of $\hat{D}$ [15], but here we will show a perhaps easier method, which is to infer these coefficients from Equation (11).

This is perhaps easiest shown by way of example. From Equation (11), and calculating the repeated derivatives of 1/r, we find that

(12) $${\left(\hat{D}R^{\left(1\right)}\right)}_{\alpha }=r_{\alpha }$$

(13) $${\left(\hat{D}R^{\left(2\right)}\right)}_{\alpha \beta }=r_{\alpha }r_{\beta }-\frac{r^{2}}{3}\delta_{\alpha ,\beta }$$

(14) $${\left(\hat{D}R^{\left(3\right)}\right)}_{\alpha \beta \gamma }=r_{\alpha }r_{\beta }r_{\gamma }-\frac{r^{2}}{5}\left(r_{\alpha }\delta_{\beta ,\gamma }+r_{\beta }\delta_{\alpha ,\gamma }+r_{\gamma }\delta_{\alpha ,\beta }\right)$$

(15) $${\left(\hat{D}R^{\left(4\right)}\right)}_{\alpha \beta \gamma \delta }=r_{\alpha }r_{\beta }r_{\gamma }r_{\delta }-\frac{r^{2}}{7}\left(r_{\alpha }r_{\beta }\delta_{\gamma ,\delta }+\ldots \right)+\frac{r^{4}}{35}\left(\delta_{\alpha \beta }\delta_{\gamma \delta }+\ldots \right)$$

where the term in brackets corresponds to a sum over all distinct permutations of the indices.

We can now define the action of the $\hat{D}$ operator on a general tensor, $A^{\left(n\right)}$, through substituting components of $A^{\left(n\right)}$ for those of $R^{\left(n\right)}$, which gives

(16) $${\left(\hat{D}A^{\left(1\right)}\right)}_{\alpha }=A_{\alpha }$$

(17) $${\left(\hat{D}A^{\left(2\right)}\right)}_{\alpha \beta }=A_{\alpha \beta }-\frac{1}{3}\underset{\chi }{\sum^{}}A_{\chi \chi }\delta_{\alpha ,\beta }$$

(18) $${\left(\hat{D}A^{\left(3\right)}\right)}_{\alpha \beta \gamma }=A_{\alpha \beta \gamma }-\frac{1}{5}\underset{\chi }{\sum^{}}\left(A_{\chi \chi \alpha }\delta_{\beta ,\gamma }+A_{\chi \chi \beta }\delta_{\alpha ,\gamma }+A_{\chi \chi \gamma }\delta_{\alpha ,\beta }\right)$$

(19) $${\left(\hat{D}A^{\left(4\right)}\right)}_{\alpha \beta \gamma \delta }=A_{\alpha \beta \gamma \delta }-\frac{1}{7}\underset{\chi }{\sum^{}}\left(A_{\chi \chi \alpha \beta }\delta_{\gamma ,\delta }+\ldots \right)+\frac{1}{35}\underset{\chi_{1}\chi_{2}}{\sum^{}}A_{\chi_{1}\chi_{1}\chi_{2}\chi_{2}}\left(\delta_{\alpha \beta }\delta_{\gamma \delta }+\ldots \right).$$

We will now show that the $\hat{D}A^{\left(n\right)}$ are traceless. Starting from a result from Applequist [15] (Section 3.3) that it always possible to find $r^{i}$ vectors such that any degree n tensor can be expressed as a linear combination in $R^{i\left(n\right)}$, viz

(20) $$A^{\left(n\right)}=\underset{i}{\sum^{}}C_{i}R^{i\left(n\right)}$$

We then apply the $\hat{D}$ operator to both sides of the above to give

(21) $$\hat{D}A^{\left(n\right)}=\underset{i}{\sum^{}}C_{i}\hat{D}R^{i\left(n\right)}$$

and given that every $\hat{D}R^{i\left(n\right)}$ is traceless we have that $\hat{D}A^{\left(n\right)}$ must also be traceless, which was to be shown.

To show that $\hat{D}$ is a proection operator onto the subspace of traceless tensors we first note that every term in ${\left(\hat{D}R^{\left(n\right)}\right)}_{\alpha \beta \gamma ..}$ in Equations (12)–(15) is of the form

(22) $$Cr^{2m}r_{\alpha }r_{\beta }..\delta_{\gamma ,\delta }\delta_{\epsilon ,\eta }..,$$

where *C* is some constant and where it is easy to show that there must be *m* Kronecker deltas if the term is to be dimensionally correct. That each term must be in this form can be shown from induction on the repeated directional derivatives of 1/r.

Looking again at Equations (12)–(15), we also see that, for each degree n, there is always a first term with *m* = 0, i.e., containing no factors of $r^{2}$, or Kronecker deltas, which is given by $r_{\alpha }r_{\beta }r_{\gamma }..$. Again, this can be shown from induction.

Now, consider what happens when we make the substitution from $R^{\left(n\right)}$ to $A^{\left(n\right)}$ in expression 22, which gives

(23) $$C\underset{\chi_{1},\chi_{2}..\chi_{m}}{\sum^{}}A_{\chi_{1}\chi_{1}\chi_{2}\chi_{2}..\chi_{m}\chi_{m}..\alpha \beta ..}^{\left(n\right)}\delta_{\gamma ,\delta }\delta_{\epsilon ,\eta }..,$$

where the sum involves taking *m* traces of $A^{\left(n\right)}$. Then if $A^{\left(n\right)}=A^{t\left(n\right)}$ is a traceless tensor, the above must be equal to zero for every term except the *m* = 0 term, which is equal to $A_{\alpha \beta \gamma ..}^{t\left(n\right)}$. Furthermore, it follows that $\hat{D}A^{t\left(n\right)}=A^{t\left(n\right)}$.

In general, we have that $A^{\left(n\right)}$ is not traceless, but given that, for any $A^{\left(n\right)}$, we have already shown that $\hat{D}A^{\left(n\right)}$ is traceless, we also have that $\hat{D}\left(\hat{D}A^{\left(n\right)}\right)=\hat{D}A^{\left(n\right)}$, and so $\hat{D}\hat{D}=\hat{D}$. It follows that $\hat{D}$ is the detracing projection operator, which projects onto the entire subspace of traceless tensors.

### 2.3. The Multipole Interaction in Terms of Traceless Tensors

In this section, we will show how the multipole interaction can be written in terms of traceless tensors.

We begin with an observation that If $A^{\left(n\right)}$ is a rank *n* tensor and $B^{t\left(n\right)}$ is a traceless tensor of the same rank, then the inner product $\langle A^{\left(n\right)},B^{t\left(n\right)}\rangle_{t}=\langle \hat{D}A^{\left(n\right)},B^{t\left(n\right)}\rangle_{t}$.

To see this, write $A^{\left(n\right)}=A^{t\left(n\right)}+A^{{}^{\prime }\left(n\right)}$, where $A^{t\left(n\right)}=\hat{D}A^{\left(n\right)}$ is the traceless part of $A^{\left(n\right)}$, and $A^{{}^{\prime }\left(n\right)}$ is the non-traceless remainder, then $\langle A^{{}^{\prime }\left(n\right)},B^{t\left(n\right)}\rangle_{t}=0$, because $A^{{}^{\prime }\left(n\right)}$ is completely outside the traceless subspace as a result of this decomposition, by construction, and the result follows.

We will now show that the traces of the multipole tensors make no contribution to the energies forces and torques, and so can be set to zero if desired.

Given that $\phi^{\left(n\right)}\left(r\right)$ is traceless, we can use the above theorem to show that the inner product in Equation (6) can be written as either $\langle M^{\left(n\right)},\phi^{\left(n\right)}\left(r\right)\rangle_{t}=\langle M^{t\left(n\right)},\phi^{\left(n\right)}\left(r\right)\rangle_{t}$, and similarly, the inner product in Equation (7) can also be written using either $M^{\left(n\right)}$ or $M^{t\left(n\right)}$, without making any difference to the result.

We have shown that the energy of a multipole in a background field is independent of its trace, and the field *from* a multipole, as given by Equation (7) is also independent of the multipole traces. Thus, the energies of any set of interacting multipoles are also independent of their traces, and the same is true for all the forces and torques being that they involve gradients of the energy.

Finally, we will show that the electrostatic potential from a multipole distribution at a given point in space can be written in terms of the traceless tensors $R^{t\left(n\right)}$ and $M^{t\left(n\right)}$. Substitution of Equation (11) into Equation (7) gives

(24) $$\phi \left(r\right)=\overset{\infty }{\underset{n=0}{\sum^{}}}\frac{\left(2n-1\right)!!}{r^{2n+1}}\langle M^{\left(n\right)},R^{t\left(n\right)}\rangle_{t}.$$

Furthermore, given the claim at the start of this section, we could use the substitutions ${\left\langle M^{\left(n\right)},R^{t\left(n\right)}\right\rangle }_{t}={\left\langle M^{t\left(n\right)},R^{t\left(n\right)}\right\rangle }_{t}={\left\langle M^{t\left(n\right)},R^{\left(n\right)}\right\rangle }_{t}$ in the above.

### 2.4. The Multipole Interaction Generating Formula

The $\nabla^{\left(n\right)}\left\{1/r\right\}$ gradients play a central role in the theory of multipoles, as should already be evident from the last section. However, as shown by Smith, the Ewald sum analogue of the multipole interaction requires the calculation of more general gradients, in which spherically symmetric functions, here written as B(r), are substituted for the 1/r terms in equalities like Equation (7). That is, instead of calculating the $\nabla^{\left(n\right)}\left\{1/r\right\}$ gradients, we will now be interested in finding gradients $\nabla^{\left(n\right)}\left\{B_{0}\left(r\right)\right\}$, with the knowledge that, should we wish to find results for the non-Ewald regular multipole expansion then we can simply set B(r)=1/r in the final expressions.

In particular, the Ewald sum analogue to the normal multipole formulae requires using the kernel B(r)=erfc(αr)/r, where erfc(x) is the complimentary error function, which corresponds to the interaction of a unit charge with a negative Gaussian ‘screening’ density, where the screening density is of the form $\rho \left(r\right)=-Aexp\left(-a^{2}r^{2}\right)$ (suitably normalised).

To simplify these gradient calculations, Smith defines the radial functions, $B_{m}\left(r\right)$, where the zeroth term is given by $B_{0}\left(r\right)=B\left(r\right)$, and with the higher order terms defined according to

(25) $$B_{m+1}\left(r\right)=-B_{m}^{\prime }\left(r\right)/r$$

from which it follows that if $B_{0}\left(r\right)=1/r$, then $B_{1}\left(r\right)=1/r^{3}$, $B_{2}\left(r\right)=3/r^{5}$, and, in general, $B_{m}\left(r\right)=\left(2m-1\right)!!/r^{2m+1}$, where !! is the double factorial.

As might be expected, the expressions are somewhat more complicated when the $B_{0}\left(r\right)=erfc\left(\alpha r\right)/r$ kernel is used, but the repeated derivatives for these functions can be readily evaluated in terms of Gaussian functions.

It is instructive to calculate the first few terms in the directional gradients of the $B_{0}\left(r\right)$ functions. Begin by recalling that for a general spherically symmetric function, f(r), we have that $\partial f\left(r\right)/\partial r_{\alpha }=f^{\prime }\left(r\right)r_{\alpha }/r$. It then follows that

(26) $$\frac{\partial }{\partial r_{\alpha }}B_{m}\left(r\right)=-r_{\alpha }B_{m+1}\left(r\right)$$

from which the first two directional derivatives evaluate to

(27) $$\frac{\partial B_{0}\left(r\right)}{\partial r_{\alpha }}=-B_{1}\left(r\right)r_{\alpha }\left(=-\frac{r_{\alpha }}{r^{3}}\right)$$

and

(28) $$\frac{\partial^{2}B_{0}\left(r\right)}{\partial r_{\alpha }r_{\beta }}=B_{2}\left(r\right)r_{\alpha }r_{\beta }-B_{1}\left(r\right)\delta_{\alpha ,\beta }\left(=\frac{1}{r^{5}}\left(3r_{\alpha }r_{\beta }-\delta_{\alpha ,\beta }r^{2}\right)\right)$$

where the terms in brackets are the results for the particular choice of $B_{0}\left(r\right)=1/r$, and where we note that these terms can be written in terms of the Maxwell Cartesian spherical harmonics we encountered in Equation (11).

It is of course possible to continue calculating higher-order terms in this fashion, but the main point we want to make here is that the first two directional derivatives of $B_{0}\left(r\right)$ above contain terms in $B_{l}\left(r\right)$, for some value of l; in general, as a consequence of the chain rule, it can be shown that gradient terms in every order can be expressed as a series in $B_{l}\left(r\right)$, where the lth derivative requires calculated functions up to the lth term.

Returning to the multipole problem, suppose that there are two multipole sites, i and j, at locations $r^{i}$ and $r^{j}$ respectively, each of which carry a set of multipoles of different ranks, where the multipoles are placed at the site locations. Then the electrostatic energy of this pair is due to each multipole on site i interacting with every multipole on site j, which can be determined by first evaluating the field at $r^{j}$ from the multipoles at $r^{i}$ according to Equation (7), and then calculating the energy of the multipoles on j in the presence of that field according to Equation (5). Labelling this energy $U^{ji}\left(r^{ji}\right)$, we obtain

(29) $$U^{ji}\left(r^{ji}\right)=\overset{\infty }{\underset{d_{j}=0}{\sum^{}}}{\left\langle M^{j\left(d_{j}\right)},\nabla_{j}^{\left(d_{j}\right)}\right\rangle }_{t}\overset{\infty }{\underset{d_{i}=0}{\sum^{}}}{\left(-1\right)}^{d_{i}}{\left\langle M^{i\left(d_{i}\right)},\nabla_{j}^{\left(d_{i}\right)}\right\rangle }_{t}\left\{B_{0}\left(r^{ji}\right)\right\}$$

where $r^{ji}=r^{j}-r^{i}$ is the inter-site vector, $r^{ji}=\left|r^{ji}\right|$, $\nabla_{j}^{\left(d_{i}\right)}$ are the directional derivatives with respect to $r^{j}$ and where we have used the more general form containing the $B_{0}\left(r^{ji}\right)$ kernel.

Similarly to how we found that every order term in the repeated derivatives of $B_{0}\left(r\right)$ contain a series in $B_{l}\left(r\right)$, it is not difficult to see that the same must be true for the interaction energy, $U^{ji}\left(r^{ji}\right)$, and, following Smith, we can make this clear by collecting terms in $B_{l}\left(r^{ji}\right)$ to write

(30) $$U^{ji}\left(r^{ji}\right)=\overset{\infty }{\underset{l=0}{\sum^{}}}B_{l}\left(r^{ji}\right)G_{ji}^{l}\left(r^{ji}\right)$$

where the $G_{ji}^{l}\left(r^{ji}\right)$ functions can be thought of as coefficients in $B_{l}\left(r^{ji}\right)$.

Evaluating these functions would seem to require calculating and summing over the $\nabla^{\left(n\right)}\left\{B\left(r\right)\right\}$ terms ‘by hand’, as it were, which would involve much tedious algebra. However, a closed form solution has been given by Lin [7], (cf. equation 4.3 in Lin’s paper) and we simply state without proof that it is given by

(31) $$G_{ji}^{l}\left(r^{ji}\right)=\underset{d_{i}+d_{c}+d_{j}=l}{\sum^{}}C_{d_{i},d_{c},d_{j}}\langle \left(M^{i\left(d_{i}+d_{c}\right)}.d_{i}.R^{ji\left(d_{i}\right)}\right),\left(M^{j\left(d_{j}+d_{c}\right)}.d_{j}.R^{ji\left(d_{j}\right)}\right)\rangle_{t},$$

where $R^{\left(n\right)}=rrr..$ (see Section 4.1); the multipoles are all assumed to be traceless; the notation A.d.B indicates a d-fold contraction over the tensor indices of A and B (again, see Section 4.1); $d_{i}$ is the number of contractions in the bracket containing $M^{i}$; $d_{j}$ is the number of contractions in the bracket containing $M^{j}$; $d_{c}$ is the number of contractions acting in the centre, between the two brackets, which we have expressed as an inner product; and where $C_{d_{i},d_{c},d_{j}}$ are integer combinatorial coefficients, given by

(32) $$C_{d_{i},d_{c},d_{j}}={\left(-1\right)}^{d_{j}}\frac{\left(d_{c}+d_{i}\right)!\left(d_{c}+d_{j}\right)!}{d_{i}!d_{c}!d_{j}!}.$$

We also have that the sum in Equation (31) is over all $d_{i},d_{j},d_{c}$, where $d_{i}+d_{c}+d_{j}=l$; $d_{i},d_{c},d_{j}\geq 0$, i.e., the sum is over all possible terms having l contractions.

We will refer to Equation (31) as the *multipole interaction generating formula*, as it generates all the terms in the multipole-multipole expansion of the electrostatic energy. Its derivation is not too difficult, if a little tedious, and essentially involves expanding out the repeated derivatives of the $B_{0}\left(r\right)$ functions, and using combinatorics to find the number of symmetrically equivalent terms.

The $G_{ji}^{l}\left(r\right)$ functions up to rank 3 are given in Section 4.1, from which it is readily apparent that the total number of dots, i.e., the number of contractions, in each term of the lth $G_{ji}^{l}\left(r^{ji}\right)$ function is equal to l, which is also apparent from the structure of Equation (31).

**Claim** **1.** *The traces of both the M tensors and the R tensors make no contribution to the multipole interaction generating formula.*

**Sketch of Proof:** For the multipoles, this follows straightforwardly from the trace condition that all multipole tensors must be traceless. Furthermore, that the same applies to the R tensors follows from the fact that their indices are completely contracted with the traceless M tensors, and then using a similar argument to that used in Section 1 for the traceless multipoles. Q.E.D.

We have shown that it is possible to detrace the R tensors in the sum of Equation (31). However, we will generally prefer to work with the tensors in their simpler, untraced, form, unless we have cause otherwise.

One of the real advantages of the Smith and Lin method for deriving the multipole interactions is that it provides a clean separation between the $G_{ji}^{l}\left(r^{ji}\right)$ functions, which depend on the traceless tensor components, and the spherically symmetric kernel dependent part, which are described by the $B_{l}\left(r^{ji}\right)$ functions. This suggests that the Smith and Lin approach may provide a useful starting point for converting the multipole interaction into spherical harmonics, and later on, we shall show how this can be done.

### 2.5. The Diagrammatic Representation

**Figure 1:**
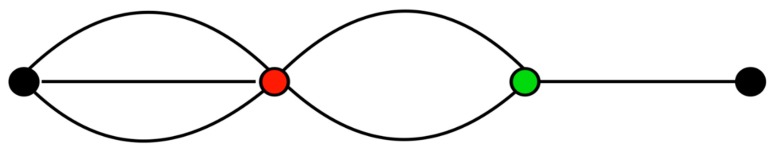
A term in the multipole interaction generating formula for l=6, corresponding to $\left(M^{i\left(5\right)}\vdots R^{ji\left(3\right)}\right):\left(M^{j\left(3\right)}.R^{ji\left(1\right)}\right)$, where the nodes from left to right represent $R^{ji}$ (black circle), $M^{i}$ (red circle), $M^{j}$ (green circle) and $R^{ji}$ (black circle).

It is possible to construct a diagrammatic representation of the multipole interaction generating formula of Equation (31), where one such diagram is shown in Figure 1. The figure shows one term in the formula for l=6, corresponding to $\left(M^{i\left(5\right)}\vdots R^{ji\left(3\right)}\right):\left(M^{j\left(3\right)}.R^{ji\left(1\right)}\right)$. The rules being that each node represents a different tensor, where its rank is given by the number of spokes radiating from the node in question; the number of spokes shared between two nodes is equal to the degree of the contraction acting between the corresponding tensors; and the sign of the diagram is taken to be negative when there are an odd number of bonds connecting the j multipole with its R tensor.

**Theorem** **1.** *The complete expression for the lth $G_{ji}^{l}\left(r^{ji}\right)$ term involves the sum over all l-bond diagrams, where the spokes on each multipole tensor are treated as distinct, and where no tensor is allowed to bond to itself.*

**Proof.**  The total number of bonds is equal to the total number of contractions which is given by $d_{i}+d_{c}+d_{j}=l$, which is the range of the sum in Equation (31). Assume that the spokes on each multipole are distinct, such that it is possible to label the spokes on both multipoles. Then the $C_{d_{i},d_{c},d_{j}}$ integer coefficients of Equation (32) are seen to be the number of unique ways of arranging the spokes on both the i and j multipoles for the topology described by contractions $d_{i},d_{c},d_{j}$. Furthermore, the negative sign mentioned above is a consequence of the ${\left(-1\right)}^{d_{j}}$ factor in Equation (32). Q.E.D. □

We also stated that no tensor may bond to itself. Given that a self-bond corresponds to taking the partial trace of a tensor, this rule follows directly from the fact that the traces of the M and R tensors make no contribution to the multipole interaction generating formula, as established in the last section.

The diagrammatic representation is by no means essential for following the math in this work, but it does provide a useful mnemonic for keeping track of the various terms in the multipole interaction. Furthermore, it will be interesting to see whether such a simple picture can still be constructed when we come to convert these expressions into spherical harmonics.

### 2.6. Forces, Fields, Angular Derivatives and Torques

We are generally interested in more than just calculating the total energy, and for completeness, this section will detail how to calculate the forces, multipole fields, angular derivatives and torques, all of which are commonly required when implementing multipole interactions into molecular-simulation code.

We begin with the forces. Let $F^{ji}\left(r_{ji}\right)=-F^{ij}\left(r_{ji}\right)$ be the force on multipole site j due to its interaction with multipoles site i, where the total force on j is given by $F^{j}=\overset{N}{\underset{i}{\sum }}F^{ji}\left(r^{ji}\right)$.

The force is found through taking the gradient of Equation (30) and evaluates to

(33) $$F^{ji}\left(r^{ji}\right)=-\frac{\partial }{\partial r^{j}}U^{ji}\left(r^{ji}\right)=r^{ji}\overset{\infty }{\underset{l=0}{\sum^{}}}B_{l+1}\left(r^{ji}\right)G_{ji}^{l}\left(r^{ji}\right)-\overset{\infty }{\underset{l=0}{\sum^{}}}B_{l}\left(r^{ji}\right)\frac{\partial }{\partial r^{j}}G_{ji}^{l}\left(r^{ji}\right)$$

where we have used Equation (26) to find the gradient of the $B_{l}\left(r^{ji}\right)$ function, and where the $\partial G_{ji}^{l}\left(r^{ji}\right)/\partial r^{j}$ functions are found from taking the gradient of Equation (31), and are given by

(34) $$\begin{array}{ll} \frac{\partial }{\partial r^{j}}G_{ji}^{l}\left(r^{ji}\right)= & \sum_{d_{i}+d_{c}+d_{j}=l}C_{d_{i},d_{c},d_{j}}[d_{i}\left(M^{i\left(d_{i}+d_{c}\right)}.d_{i}-1.R^{ji\left(d_{i}-1\right)}\right).d_{c}.\left(M^{j\left(d_{j}+d_{c}\right)}.d_{j}.R^{ji\left(d_{j}\right)}\right)\\ & +d_{j}\left(M^{i\left(d_{i}+d_{c}\right)}.d_{i}.R^{ji\left(d_{i}\right)}\right).d_{c}.\left(M^{j\left(d_{j}+d_{c}\right)}.d_{j}-1.R^{ji\left(d_{j}-1\right)}\right)] \end{array}.$$

The $\partial G_{ji}^{l}\left(r^{ji}\right)/\partial r^{j}$ functions up to rank 3 are listed in Section 4.2.

**Figure 2:**
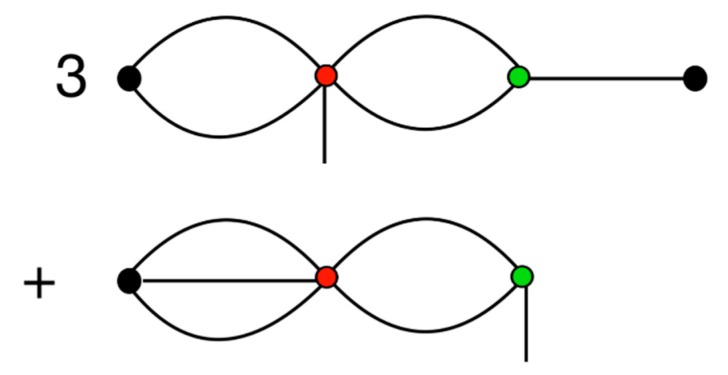
A force term obtained from taking the gradient of the term in Figure 1, which equates to $3\left(M^{i\left(5\right)}:R^{ji\left(2\right)}\right):\left(M^{j\left(3\right)}.R^{ji\left(1\right)}\right)+\left(M^{i\left(5\right)}\vdots R^{ji\left(3\right)}\right):M^{j\left(3\right)}$.

A diagrammatic representation of one force term is shown in Figure 2, which corresponds to taking the gradient of the interaction from Figure 1. Comparing Figure 1 and Figure 2, it can be seen that taking the gradient results in breaking one of the bonds connecting either the i or j multipole with its R tensor. This leaves the multipole with a bare (or unbonded) spoke, which means that, taken as a whole, we have a diagrammatic representation of a rank 1 vector.

We have already provided an expression for the total energy of the system, but an arguably more elegant expression for the energy is in terms of multipole field tensors, according to

(35) $$U=\frac{1}{2}\overset{N}{\underset{i}{\sum^{}}}\overset{\infty }{\underset{n=0}{\sum^{}}}{\left\langle M^{i\left(n\right)},\phi^{i\left(n\right)}\right\rangle }_{t},$$

where $\phi^{i\left(n\right)}$ is the rank *n* field tensor on site i, which is defined from

(36) $$\phi^{i\left(n\right)}=\frac{\partial U}{\partial M^{i\left(n\right)}}$$

Equation (35), which is written in terms of multipole fields has the distinct advantage that it allows for an automatic decomposition of the total energy into contributions from the different multipole ranks, i.e., charge, dipole, quadrupole etc. Furthermore, another reason why we may want to calculate the fields is that it greatly simplifies calculation of angular derivatives and torques, to be given in the next section.

The fields can be calculated by finding the derivative of the energy with respect to each multipole. Looking at just one pair of multipole sites, the field on multipole site j due to the multipoles on site i is given by

(37) $$\phi^{ji\left(n\right)}=\frac{\partial U^{ji}\left(r^{ji}\right)}{\partial M^{j\left(n\right)}}=\overset{\infty }{\underset{l=0}{\sum^{}}}B_{l}\left(r^{ji}\right)\frac{\partial }{\partial M^{j\left(n\right)}}G_{ji}^{l}\left(r^{ji}\right)$$

where, taking the derivative of Equation (31) with respect to the multipoles we obtain

(38) $$\frac{\partial }{\partial M^{j\left(n\right)}}G_{ji}^{l}\left(r^{ji}\right)=\mathrm{SYMM}\left[\underset{\underset{d_{c}+d_{j}=n}{d_{i}+d_{c}+d_{j}=l}}{\sum^{}}C_{d_{i},d_{c},d_{j}}\left(M^{i\left(d_{i}+d_{c}\right)}.d_{i}.R^{ji\left(d_{i}\right)}\right)R^{ji\left(d_{j}\right)}\right]$$

where SYMM indicates that the resulting tensor elements are to be symmetrised over all index permutations, and for their traces to be removed.

This final step is necessary to ensure that the field tensors have the same symmetries as their corresponding multipoles, given that per their definition, the field tensors must be unchanged with respect to any permutation of their indices. Furthermore, the removal of the traces is because the field traces make no contribution to the energy, and so it makes sense that these are set to zero.

**Figure 3:**
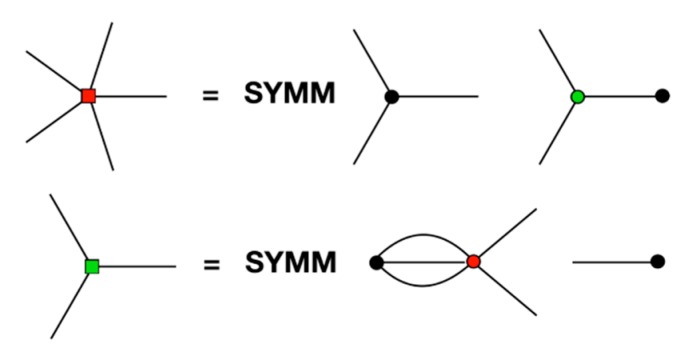
Representation of the multipole field terms for the interaction in Figure 1. The spokes radiating from coloured squares represent multipole fields tensors, of rank given by their number of spokes. The star with the central red square represents the rank-5 field tensor on the i site, and the star with the central green square represents the rank-3 field tensor on the j site.

A diagrammatic representation of the field calculation is shown in Figure 3, in which the rank 5 field on $M^{i\left(5\right)}$ and the rank 3 field on $M^{j\left(3\right)}$, corresponding to the multipole interaction in Figure 1, are shown. Both diagrams can be thought of as cutting a multipole free from Figure 1, which corresponds to taking the derivative with respect to that multipole.

In order that we may calculate the angular derivatives of the energy, let $O_{\alpha \beta }$ be orthogonal rotation matrices which transform vector components from the reference frame to the laboratory frame (see Section 4.1). Furthermore, suppose that $O_{\alpha \beta }=O_{\alpha \beta }\left(\phi ,\theta ,\psi \right)$, where (ϕ,θ,ψ) are (three) Euler angles (e.g., see ref. [19]) and we wish to find the energy derivatives with respect to these angles. Taking the Euler angle ψ as an example, use the chain rule to obtain the contribution from the rank n multipole as

(39) $$\begin{array}{c} \frac{\partial U}{\partial \psi }=\sum_{n=1}^{\infty }\sum_{\alpha \beta \gamma ..}\frac{\partial U}{\partial M_{\alpha \beta \gamma ..}^{\left(n\right)}}\frac{\partial M_{\alpha \beta \gamma ..}^{\left(n\right)}}{\partial \psi }\\ =\sum_{n=1}^{\infty }n\sum_{\alpha \beta \gamma ..}\phi_{\alpha \beta \gamma ..}^{\left(n\right)}\sum_{\alpha^{\prime }\beta^{\prime }\gamma^{\prime }..}\frac{\partial }{\partial \psi }\left\{O_{\alpha \alpha^{\prime }}\right\}O_{\beta \beta^{\prime }}O_{\gamma \gamma^{\prime }}..M_{\alpha^{\prime }\beta^{\prime }\gamma^{\prime }..}^{ref\left(n\right)} \end{array}$$

where we have used Equation (78) from Section 4.1 to relate the components of the multipole tensor to their components in the reference frame, and where the factor of n in the above derives from the index permutation symmetry of $\phi_{\alpha \beta \gamma ..}^{\left(n\right)}$ and $M_{\alpha \beta \gamma ..}^{ref\left(n\right)}$, such that it can be shown that each of the *n* terms involving derivatives of the rotation matrices are identical, and so can be added together.

The torques may be evaluated from the energy derivatives with respect to rotations about each axis. First consider an infinitesimal rotation by $\Delta \theta_{\alpha }$ about the α axis. If a vector r has the value $r\left(\Delta \theta_{\alpha }\right)$ after the rotation, then we have $r\left(\Delta \theta_{\alpha }\right)=r+\Delta \theta_{\alpha }\hat{\alpha }\times r$, which has components

(40) $$r_{\beta }\left(\Delta \theta_{\alpha }\right)=r_{\beta }+\Delta \theta_{\alpha }\underset{\delta }{\sum^{}}\epsilon_{\beta \alpha \delta }r_{\delta }$$

where $\epsilon_{\alpha \beta \gamma }$ is the Levi-Civita symbol, $\epsilon_{xyz}=\epsilon_{yzx}=\epsilon_{zxy}=1$, $\epsilon_{zyx}=\epsilon_{yxz}=\epsilon_{xzy}=-1$, and ϵ=0 otherwise.

It will prove useful to recast Equation (40) in terms of a rotation matrix. We have $r_{\beta }\left(\Delta \theta_{\alpha }\right)=\sum_{\gamma }O_{\beta \gamma }\left(\Delta \theta_{\alpha }\right)r_{\gamma }$, where $O_{\beta \gamma }\left(\Delta \theta_{\alpha }\right)$ is given by

(41) $$O_{\beta \gamma }\left(\Delta \theta_{\alpha }\right)=\delta_{\beta ,\gamma }+\Delta \theta_{\alpha }\epsilon_{\beta \alpha \gamma }$$

The components of the torque, $t_{\alpha }$, are obtained from substitution of the above into Equation (39) to give

(42) $$t_{\alpha }=-\frac{\partial U}{\partial \theta_{\alpha }}{|}_{\theta_{\alpha }=0}=-\overset{\infty }{\underset{n=1}{\sum^{}}}n\underset{\beta \gamma \delta \epsilon \nu ..}{\sum^{}}\epsilon_{\alpha \beta \gamma }M_{\delta \epsilon \nu ..\beta }^{\left(n\right)}\phi_{\delta \epsilon \nu ..\gamma }^{\left(n\right)}$$

where we have used $\partial O_{\beta \gamma }\left(\theta_{\alpha }\right)/\partial \theta_{\alpha }=\epsilon_{\beta \alpha \gamma }$, and $O_{\beta \gamma }\left(0\right)=\delta_{\beta ,\gamma }$, both of which can be deduced from Equation (41).

**Figure 4:**
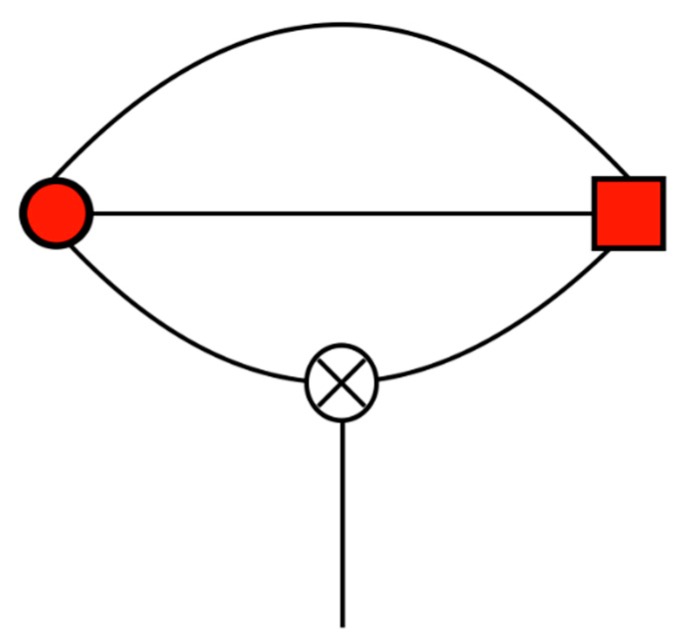
The (negative of) the torque on a rank-3 multipole. The red circle represents the multipole, the red square is its rank-3 field, and the circle with the cross represents a rank-1 vector cross product.

A diagrammatic representation of the torque for one multipole is given in Figure 4.

### 2.7. Polynomials and Symmetric Tensors

This section explores the deep connection between homogeneous polynomials and symmetric tensors, covering similar ground to the treatment by Applequist [15].

We begin with introducing some useful notation. Suppose that $A_{\alpha \beta \gamma ..}^{\left(n\right)}$ is a symmetric Cartesian tensor component where the αβγ.. index contains $n_{x}$ occurrences of x, $n_{y}$ occurrences of y and $n_{z}$ occurrences of z. Defining $n=\left(n_{x},n_{y},n_{z}\right)$, it will be useful to introduce what Applequist refers to as compressed tensor notation, in which we write

(43) $$A_{\alpha \beta \gamma ..}^{\left(n\right)}=A_{\beta \alpha \gamma ..}^{\left(n\right)}=A_{\beta \gamma \alpha ..}^{\left(n\right)}=..=A_{\left(n_{x},n_{y},n_{z}\right)}^{\left(n\right)}=A_{n}^{\left(n\right)}$$

where, in total, there is a multinomial of $n!/\left(n_{x}!n_{y}!n_{z}!\right)$ permutations of the αβγ.. indices belonging to a particular n.

Now suppose that $p^{\left(n\right)}\left(r\right)$ is a homogeneous polynomial of degree n, such that $p^{\left(n\right)}\left(\lambda r\right)=\lambda^{n}p^{\left(n\right)}\left(r\right)$, where λ is a scalar. For example, a degree 3 homogenous polynomial is given by $p^{a\left(3\right)}\left(r\right)=4x^{3}+2y^{2}x-5y^{3}$.

To express $p^{\left(n\right)}\left(r\right)$ in general form, we use compressed notation to write $R_{n}^{\left(n\right)}=x^{n_{x}}y^{n_{y}}z^{n_{z}}$, and we will label the monomial coefficient in the $R_{n}^{\left(n\right)}$ component as ${\bar{P}}_{n}^{\left(n\right)}$. The bar is used to signify that the ${\bar{P}}_{n}^{\left(n\right)}$ are monomial coefficients, although we will shortly see that the ${\bar{P}}_{n}^{\left(n\right)}$ can also be interpreted as tensor components.

In terms of the polynomial coefficients ${\bar{P}}_{n}^{\left(n\right)}$, we can express any degree n homogeneous polynomial as

(44) $$p^{\left(n\right)}\left(r\right)=\underset{\left|n\right|=n}{\sum^{}}{\bar{P}}_{n}^{\left(n\right)}R_{n}^{\left(n\right)}=\underset{\alpha \beta \gamma ..}{\sum^{}}P_{\alpha \beta \gamma ..}^{\left(n\right)}R_{\alpha \beta \gamma ..}^{\left(n\right)},$$

where the sum is over all values of $n=\left(n_{x},n_{y},n_{z}\right)$ for which $n_{x}+n_{y}+n_{z}=n$, and where we have also introduced the symmetric Cartesian tensor, $P^{\left(n\right)}$.

To clarify the above, it may help to take an example. The second order polynomial, $p^{\left(2\right)}\left(r\right)$, can be written as either

(45) $$p^{\left(2\right)}\left(r\right)={\bar{P}}_{\left(2,0,0\right)}^{\left(2\right)}x^{2}+{\bar{P}}_{\left(0,2,0\right)}^{\left(2\right)}y^{2}+{\bar{P}}_{\left(0,0,2\right)}^{\left(2\right)}z^{2}+{\bar{P}}_{\left(1,1,0\right)}^{\left(2\right)}xy+{\bar{P}}_{\left(1,0,1\right)}^{\left(2\right)}xz+{\bar{P}}_{\left(0,1,1\right)}^{\left(2\right)}yz$$

or

(46) $$p^{\left(2\right)}\left(r\right)=P_{xx}^{\left(2\right)}x^{2}+P_{yy}^{\left(2\right)}y^{2}+P_{zz}^{\left(2\right)}z^{2}+2P_{xy}^{\left(2\right)}xy+2P_{xz}^{\left(2\right)}xz+2P_{yz}^{\left(2\right)}yz$$

where we have used the permutation symmetry of $P^{\left(2\right)}$.

Equating terms in the polynomial, we have ${\bar{P}}_{\left(2,0,0\right)}^{\left(2\right)}=P_{xx}^{\left(2\right)}$, and ${\bar{P}}_{\left(1,1,0\right)}^{\left(2\right)}=2P_{xy}^{\left(2\right)}$, and similarly for other terms. In general, $P_{\alpha \beta \gamma ..}^{\left(n\right)}$ is related to ${\bar{P}}_{n}^{\left(n\right)}$ by

(47) $$P_{\alpha \beta \gamma ..}^{\left(n\right)}=\frac{n_{x}!n_{y}!n_{z}!}{n!}{\bar{P}}_{n}^{\left(n\right)}$$

where the inverse multinomial coefficients are required due to the permutation symmetry of the αβγ.. indices.

We can also form inner products, ${\left\langle P^{\left(n\right)},A^{\left(n\right)}\right\rangle }_{t}$, which can be evaluated as

(48) $${\left\langle P^{\left(n\right)},A^{\left(n\right)}\right\rangle }_{t}=\underset{\alpha \beta \gamma ..}{\sum^{}}P_{\alpha \beta \gamma ..}^{\left(n\right)}A_{\alpha \beta \gamma ..}^{\left(n\right)}=\underset{\left|n\right|=n}{\sum^{}}{\bar{P}}_{n}^{\left(n\right)}A_{n}^{\left(n\right)}$$

Given that $P^{\left(n\right)}$ encodes all the information in the polynomial coefficients, i.e., there is a one to one mapping between the polynomial coefficients and the tensor components, we can think of $P^{\left(n\right)}$ as being the symmetric tensor form of ${\bar{P}}_{n}^{\left(n\right)}$.

Technically, $P^{\left(n\right)}$ is a so-called *covariant* tensor, which transforms under rotations in the opposite sense to $R^{\left(n\right)}$. To see why, consider a rotation of the coordinate system

(49) $$\begin{array}{l} p\left(Or\right)=\sum_{\alpha \beta \gamma }P_{\alpha \beta \gamma ..}^{\left(n\right)}\sum_{\alpha^{\prime }\beta^{\prime }\gamma^{\prime }..}O_{\alpha \beta \gamma ..,\alpha^{\prime }\beta^{\prime }\gamma^{\prime }..}^{\left(n\right)}R_{\alpha^{\prime }\beta^{\prime }\gamma^{\prime }..}^{\left(n\right)}\\ =\sum_{\alpha^{\prime }\beta^{\prime }\gamma^{\prime }..}\left(\sum_{\alpha \beta \gamma }O^{\left(n\right)}{}_{\alpha^{\prime }\beta^{\prime }\gamma^{\prime }..,\alpha \beta \gamma ..}^{T}P_{\alpha \beta \gamma ..}^{\left(n\right)}\right)R_{\alpha^{\prime }\beta^{\prime }\gamma^{\prime }..}^{\left(n\right)} \end{array}$$

where $O^{\left(n\right)}$ is the orthogonal tensor rotation matrix, $O_{\alpha \beta \gamma ..,\alpha^{\prime }\beta^{\prime }\gamma^{\prime }}^{\left(n\right)}=O_{\alpha \alpha^{\prime }}O_{\beta \beta^{\prime }}O_{\gamma \gamma^{\prime }}..$ (See Section 4.1.)

This shows that we can find p(Or) from *either* transforming the components of $R^{\left(n\right)}$ by the usual orthogonal rotation $O^{\left(n\right)}$, *or*, we can do it by transforming the components of $P^{\left(n\right)}$ by the *inverse* rotation $O^{\left(n\right)}{}^{T}=O^{\left(n\right)}{}^{-1}$.

The $P^{\left(n\right)}$ tensor may also be obtained from the polynomial itself by way of a linear operator, $\hat{L}=\left(1/n!\right)\nabla^{\left(n\right)}$, according to

(50) $$P^{\left(n\right)}=\hat{L}p^{\left(n\right)}\left(r\right)=\frac{1}{n!}\nabla^{\left(n\right)}p^{\left(n\right)}\left(r\right)$$

which may be seen by applying the above gradient operator to Equation (44). Note that because we are taking n derivatives of a degree-n homogeneous polynomial that the LHS above is independent of r.

Furthermore, we have that the inverse to Equation (50) is given by

(51) $$p^{\left(n\right)}\left(r\right)={\left\langle P^{\left(n\right)},R^{\left(n\right)}\right\rangle }_{t}.$$

We now introduce the so-called *harmonic polynomials*, $h^{\left(n\right)}\left(r\right)$, which are a subset of the $p^{\left(n\right)}\left(r\right)$ polynomials which have a vanishing Laplacian, $\nabla^{2}h^{\left(n\right)}\left(r\right)=0$. As an example, one such polynomial is given by $h^{a\left(3\right)}=2x^{3}z+3x^{2}y-6xy^{2}z-y^{3}$, for which it may be confirmed that $\Delta h^{a\left(3\right)}=0$.

The harmonic polynomials are of particular interest, as their corresponding symmetric tensors, given by $H^{\left(n\right)}=\left(1/n!\right)\nabla^{\left(n\right)}h^{\left(n\right)}\left(r\right)$, are traceless, due to the vanishing Laplacian condition on $h^{\left(n\right)}\left(r\right)$, i.e.,

(52) $$\underset{\gamma }{\sum^{}}H_{\alpha \beta ..\gamma \gamma }^{\left(n\right)}=\frac{1}{n!}\frac{\partial^{n-2}}{\partial r_{\alpha }\partial r_{\beta }..}\nabla^{2}h^{\left(n\right)}\left(r\right)=0.$$

Thus, the linear operator, $\hat{L}=\left(1/n!\right)\nabla^{\left(n\right)}$, together with its inverse in Equation (51), establishes an isomorphism between the vector spaces of harmonic polynomials of degree *n*, and symmetric traceless tensors of rank n.

The harmonic polynomials, or equivalently the rank n traceless tensors are spanned by 2*n* + 1 linearly independent vectors. To see this, first consider the components, $A_{n}$, of a rank n symmetric Cartesian tensor for which it is a simple matter to show that there are $N_{s}=\left(n+1\right)\left(n+2\right)/2$ possible values of $n=\left(n_{x},n_{y},n_{z}\right)$ for which $n_{x}+n_{y}+n_{z}=n$. Furthermore, the trace condition imposes $N_{t}=n\left(n-1\right)/2$ constraints. To see this, take the trace tensor, $Tr\left(A^{\left(n\right)}\right)$ from Equation (9), which is a degree *n* − 2 symmetric tensor, having components $Tr{\left(A^{\left(n\right)}\right)}_{\alpha \beta \gamma ..}=\sum_{\chi }A_{\alpha \beta \gamma ..\chi \chi }^{\left(n\right)}$, where clearly, each component is independent, and as such the trace tensor is described by n(n−1)/2 linearly independent vectors, all of which must be independently equal to zero in order that $A^{\left(n\right)}$ be traceless. This leaves us with $N_{st}=N_{s}-N_{t}=2n+1$ degrees of freedom for both the rank *n* traceless tensors and the degree *n* harmonic polynomials.

### 2.8. Spherical Harmonics as a Basis for Traceless Symmetric Tensors

The discussion at the end of the last section referred to the fact that symmetric traceless tensors can in principle be spanned by a minimal set of 2*n* + 1 linearly independent vectors. It would obviously be advantageous to work in a representation in which just this number of components are used, and in this section we shall show how this can be done using spherical harmonics, which provide a natural orthonormal basis for traceless tensors.

We begin by defining what we will refer to as the spherical inner product, $\langle ,\rangle_{s}$, not to be confused by our tensor inner product, $\langle ,\rangle_{t}$, which is given by

(53) $$\langle p^{a\left(m\right)},p^{b\left(n\right)})\rangle_{s}=\int p^{a\left(m\right)}\left(\phi ,\theta \right)p^{b\left(n\right)}\left(\phi ,\theta \right)dS$$

where the superscripts a and b label two homogeneous polynomials of order *m* and *n*, respectively, and we have switched to spherical coordinates. (Also note, here we are using real polynomials, but either one of $p^{a\left(n\right)}$, or $p^{b\left(n\right)}$ in the integral would need to be replaced by its complex conjugate in the full complex case, in order that $\langle ,\rangle_{s}$ be a true inner product.)

We now state a theorem which will allow us to convert between the tensor inner product of traceless tensors and the spherical inner product of harmonic polynomials.

#### A Theorem on the Equivalence of Two Inner Products

Suppose that $h^{\left(n\right)}\left(r\right)$ is a degree n harmonic polynomial, and $H^{\left(n\right)}=\left(1/n!\right)\nabla^{\left(n\right)}h^{\left(n\right)}\left(r\right)$ is its traceless tensor equivalent, then the spherical inner product, $\langle h^{a\left(n\right)},h^{b\left(n\right)}\rangle_{s}$, and the tensor inner product, $\langle H^{a\left(n\right)},H^{b\left(n\right)}\rangle_{t}$, are in a constant ratio for each rank, according to

(54) $${\left\langle h^{a\left(n\right)},h^{b\left(n\right)}\right\rangle }_{s}=\frac{4\pi n!}{\left(2n+1\right)!!}{\left\langle H^{a\left(n\right)},H^{b\left(n\right)}\right\rangle }_{t}.$$

The above theorem may seem like it would be easy to derive through standard algebraic methods, but in fact, it is surprisingly hard to obtain and our derivation ended up being quite technical. Thus, we will leave the mathematical details to Section 4.4 through Section 4.6; Section 4.5, we show that the two inner products are proportional, whilst in Section 4.6 we derive the proportionality constant. We should also note that similar expressions to Equation (54) have been developed by Ehrentraut and Muschik, [20] (especially Section 4 in this reference) although using a quite different approach to the one taken here.

From the theory of spherical harmonics, a complete orthogonal basis (with respect to the spherical inner product) for degree harmonic polynomials is provided by the *spherical harmonics* (technically, the regular solid harmonics), which comprise a set of 2n + 1 real harmonic polynomials orthogonal over the unit sphere, such that, writing the spherical harmonic polynomials as $q^{i\left(n\right)}\left(r\right)$, we have that

(55) $$\frac{\langle q^{i\left(m\right)},q^{j\left(n\right)}\rangle_{s}}{∥q^{i\left(m\right)}{∥}_{s}∥q^{j\left(n\right)}{∥}_{s}}=\delta_{i,j}\delta_{m,n}$$

where $∥q^{i\left(m\right)}{∥}_{s}=\sqrt{\langle q^{i\left(m\right)},q^{i\left(m\right)}\rangle_{s}}$ is the norm of $q^{i\left(m\right)}\left(r\right)$, and similarly for $q^{j\left(n\right)}\left(r\right)$.

A note on the nomenclature. Strictly speaking, a spherical harmonic can be used to describe *any* harmonic polynomial confined to the unit sphere. However, here we will use the term spherical harmonic polynomial to refer specifically to the set of $q^{i\left(n\right)}\left(r\right)$ polynomials, which are orthogonal over the unit sphere.

By application of Equation (50), we can also define the traceless tensor form of the spherical harmonics, which we will call $Q^{i\left(n\right)}$, from

(56) $$Q^{i\left(n\right)}=\frac{1}{n!}\nabla^{\left(n\right)}q^{i\left(n\right)}\left(r\right).$$

which, using Equation (51), has an inverse given by

(57) $$q^{i\left(n\right)}\left(r\right)={\left\langle Q^{i\left(n\right)},R^{\left(n\right)}\right\rangle }_{t}=\underset{\left|n\right|=n}{\sum^{}}{\bar{Q}}_{n}^{i\left(n\right)}R_{n}^{\left(n\right)}$$

The spherical harmonic polynomials up to rank 3 are given in Table 1, adapted from Stone [13].

Given the orthogonality of the spherical harmonic polynomials from Equation (55) and given the inner product equivalence from Equation (54), we also have that, under suitable normalisation,

(58) $${\left\langle Q^{i\left(n\right)},Q^{j\left(n\right)}\right\rangle }_{t}=\delta_{i,j}.$$

A note on the normalisation: Here, we are choosing the normalisation such that Equation (58) above holds, i.e., $∥Q^{i\left(n\right)}{∥}^{2}=1$. So that the $q^{i\left(n\right)}\left(r\right)$ are consistent with the $Q^{i\left(n\right)}$ according to Equation (56), we have, by way of Equation (54), that the $q^{i\left(n\right)}\left(r\right)$ polynomials should be normalised according to $∥q^{i\left(n\right)}{∥}^{2}=4\pi n!/\left(2n+1\right)!!$.

Thus the $Q^{i\left(n\right)}$ provide an orthogonal basis for the traceless symmetric rank n tensors, and as such, we can express any symmetric traceless rank n tensor as a linear sum in $Q^{i\left(n\right)}$, according to

(59) $$A^{\left(n\right)}=\overset{2n+1}{\underset{k=1}{\sum^{}}}A_{k}^{\left(n\right)}Q^{k\left(n\right)}$$

and taking the inner product of both sides of Equation (59) above with respect to $Q^{i\left(n\right)}$ shows that $A_{i}^{\left(n\right)}$, the components of $A^{\left(n\right)}$ in the spherical harmonic basis are given by

(60) $$A_{i}^{\left(n\right)}={\left\langle Q^{i\left(n\right)},A^{\left(n\right)}\right\rangle }_{t}=\underset{\left|n\right|=n}{\sum^{}}{\bar{Q}}_{n}^{i\left(n\right)}A_{n}^{\left(n\right)}$$

where we use the convention that spherical harmonic components are to be indexed by modern roman lower case letters, as opposed to Greek for the Cartesian indices.

Conversion of traceless tensors from Cartesians to spherical harmonics according to Equation (60) is perhaps most easily done through consulting tables of spherical harmonics polynomials, such as those given by Stone [13]. Furthermore, for convenience, the spherical harmonic polynomials up to rank 3 are listed in Table 1, which is adapted from Stone.

As an example, from Table 1 (Section 4.7), we have that the spherical harmonic $q^{4\left(3\right)}=\sqrt{3/2}z\left(x^{2}-y^{2}\right)$, which gives, $A_{4}^{\left(3\right)}=\sqrt{3/2}\left(A_{zxx}-A_{zyy}\right)$, and given that $q^{1\left(2\right)}=\left(\sqrt{6}/6\right)\left(3z^{2}-r^{2}\right)$, we have that $A_{1}^{\left(2\right)}=\left(\sqrt{6}/6\right)\left(3A_{zz}-\left(A_{xx}+A_{yy}+A_{zz}\right)\right)$, and so on.

The spherical harmonic representation comes in particularly useful for calculating inner products; for, we can use the orthogonality of spherical harmonics to write

(61) $${\left\langle A^{\left(n\right)},B^{\left(n\right)}\right\rangle }_{t}=\overset{2n+1}{\underset{i,j=1}{\sum^{}}}A_{i}^{\left(n\right)}B_{j}^{\left(n\right)}{\left\langle Q^{i\left(n\right)},Q^{j\left(n\right)}\right\rangle }_{t}=\overset{2n+1}{\underset{i=1}{\sum^{}}}A_{i}^{\left(n\right)}B_{i}^{\left(n\right)}$$

Thus, it can be seen that calculating an inner product in spherical harmonics requires the minimal 2*n* + 1 operations for that rank, which is an enormous saving over the $3^{n}$ multiplications required for naively multiplying all of the $A_{\alpha \beta \gamma ..}^{\left(n\right)}B_{\alpha \beta \gamma ..}^{\left(n\right)}$ matrix Cartesian components together [21,22].

We have seen how the $Q_{n}^{i\left(n\right)}$ coefficients allow for transformation of Cartesians into spherical harmonics. There is also an inverse transformation, given by $R_{k}^{\alpha \beta \gamma ..\left(n\right)}$, which are the components of $R_{\alpha \beta \gamma ..}^{\left(n\right)}$ projected onto the spherical harmonic basis, and which can be used to transform the components in the spherical harmonic basis back to Cartesians.

Furthermore, once again, Stone has provided tables which give a very convenient way for carrying out these transformations; Table 2, which is also adapted from Stone, provides the relevant information.

As an example, from Table 2, we have that $xy^{2}=-\left(1/2\right)q^{6\left(3\right)}-\left(\sqrt{15}/30\right)q^{2\left(3\right)}$, from which it follows that, if we have spherical harmonic components $A_{k}^{\left(3\right)}$, then $A_{xyy}^{\left(3\right)}=-\left(1/2\right)A_{6}^{\left(3\right)}-\left(\sqrt{15}/30\right)A_{2}^{\left(3\right)}$.

We conclude this section with a discussion of what Applequist [15] has called the detracing operator.

Suppose that $T^{\left(n\right)}$ is an in-general non-traceless symmetric tensor. Given that the spherical harmonics form a complete orthonormal basis for the subspace of traceless rank n tensors, the detracing operator ${\hat{D}}_{n}$ can be written in spherical harmonics as

(62) $$\hat{D}T^{\left(n\right)}=\overset{2n+1}{\underset{i=1}{\sum^{}}}{\left\langle T^{\left(n\right)},Q^{i\left(n\right)}\right\rangle }_{t}Q^{i\left(n\right)}$$

One application of $\hat{D}$ is in particular worth noting. Applying the above expression for $R^{\left(n\right)}$, and using Equation (57) to make the substitution ${\left\langle R^{\left(n\right)},Q^{i\left(n\right)}\right\rangle }_{t}=q^{i\left(n\right)}\left(r\right)$ gives via Equation (60) that

(63) $$R_{i}^{t\left(n\right)}=q^{i\left(n\right)}\left(r\right).$$

where $R^{t}\left(n\right)=\hat{D}R$ are the traceless projections of the $R^{\left(n\right)}$ tensors (see Equation (11)), and where we have used ${\left\langle R^{\left(n\right)},Q^{i\left(n\right)}\right\rangle }_{t}={\left\langle \hat{D}R^{\left(n\right)},Q^{i\left(n\right)}\right\rangle }_{t}$, which holds because only the traceless component of $R^{\left(n\right)}$ can contribute to the inner product with the traceless $Q^{i\left(n\right)}$.

Thus, the spherical harmonic components of the Maxwell Cartesian spherical harmonics are just the spherical harmonic polynomials themselves.

## 3. Discussion

### 3.1. The Multipole Interaction in Spherical Harmonics

In this section, we will aim to convert the various expressions so far developed in Cartesians into their spherical harmonic equivalents.

In the last section, we showed how to convert inner products into spherical harmonics. Furthermore, in this regard, it is unfortunate the multipole interaction generating formula of Equation (31) cannot be expressed entirely in terms of such products, involving as it does problematic contractions of the form $C^{\left(d_{c}\right)}=M^{\left(d_{i}+d_{c}\right)}.d_{i}.R^{\left(d_{i}\right)}$.

However, even when dealing with such contractions, there is a way of still using the inner product method, which we shall now describe. We introduce what we will refer to as the *split-component representation* of a symmetric tensor, using the notation $T^{\left(n_{a},n_{b}\right)}$, where $n=n_{a}+n_{b}$ is the full rank of the tensor, $T^{\left(n_{a}+n_{b}\right)}$, of which $T^{\left(n_{a},n_{b}\right)}$ is but one representation. Taking $n_{a}=3$, $n_{b}=2$ as an illustrative example, we write the symmetric traceless multipole tensor, $M^{\left(3,2\right)}$, which has Cartesian components

(64) $$M_{\alpha \beta \gamma ,\delta \epsilon }^{\left(3,2\right)}=M_{\alpha \beta \gamma \delta \epsilon }^{\left(5\right)}$$

where $M^{\left(3,2\right)}$ transforms as a symmetric traceless Cartesian tensor with respect to (i) its before-comma components, (ii) its after-comma components, and (iii) in all its components as a whole. In this representation, an example contraction can now be written as

(65) $$C_{\alpha \beta \gamma }^{\left(3\right)}=\underset{\delta \epsilon }{\sum^{}}M_{\alpha \beta \gamma \delta \epsilon }^{\left(5\right)}R_{\delta \epsilon }^{\left(2\right)}=\underset{\delta \epsilon }{\sum^{}}M_{\alpha \beta \gamma ,\delta \epsilon }^{\left(3,2\right)}R_{\delta \epsilon }^{\left(2\right)}$$

which behaves like an inner product with respect to the after-comma components, and, as such, can be readily evaluated in spherical harmonics.

We proceed by separately transforming the before-comma and after-comma components of $M_{\alpha \beta \gamma ,\delta \epsilon }^{\left(3,2\right)}$ into spherical harmonics, and, using transformations of the sort described by Equation (60), we have that

(66) $$M_{i,j}^{\left(3,2\right)}=\underset{\alpha \beta \gamma \delta \epsilon }{\sum^{}}Q_{\alpha \beta \gamma }^{i\left(3\right)}Q_{\delta \epsilon }^{j\left(2\right)}M_{\alpha \beta \gamma \delta \epsilon }^{\left(5\right)},$$

where $Q_{\delta \epsilon }^{j\left(2\right)}$ is used to transform $M_{\alpha \beta \gamma ,\delta \epsilon }^{\left(3,2\right)}\to M_{\alpha \beta \gamma ,k}^{\left(3,2\right)}$, and $Q_{\alpha \beta \gamma }^{i\left(3\right)}$ is used to transform $M_{\alpha \beta \gamma ,k}^{\left(3,2\right)}\to M_{j,k}^{\left(3,2\right)}$. (As discussed in the last section, these transformations are easiest done by way of tables of spherical harmonics, suitably implemented into code.)

Also, and referring to the discussion of the detracing operator of Equation (62), the $R^{\left(n\right)}$ tensor components are transformed as $R_{\alpha \beta }^{\left(n\right)}\to R_{i}^{t\left(n\right)}=q^{i\left(n\right)}\left(r\right)$, and so the desired contraction can now be written in spherical harmonics as

(67) $$C_{a}^{\left(3\right)}=\overset{5}{\underset{b=1}{\sum^{}}}M_{a,b}^{\left(3,2\right)}q^{b\left(2\right)}\left(r\right)$$

Notes:

(i) The concept of a split component representation can be made quite general. If we wished to, we could use a mixed Cartesian-spherical harmonic representation, such as $M_{\alpha \beta \gamma ,k}^{\left(3,2\right)}$, or we could choose to use more than one; for instance, $M_{a,b,c}^{\left(3,2,1\right)}$ is a valid split of $M^{\left(6\right)}$. However, no matter how we choose the split, or the base, it is still referring to the same underlying tensor, and, if necessary, one can always recover all the original components from the by taking the appropriate inverse transformations.
(ii) The rank-1 spherical harmonics are just x, y and z (see Table 1, in Section 4.7), from which it follows that a rank 1 tensor has spherical harmonic components $T_{a}^{\left(1\right)}=T_{x}^{\left(1\right)},T_{y}^{\left(1\right)},T_{z}^{\left(1\right)}$, the same as in Cartesians. Furthermore, in general, we have that $T_{\alpha ,\beta ,\gamma ,.}^{\left(1,1,1,..\right)}=T_{\alpha \beta \gamma ..}^{\left(n\right)}$.
(iii) The split component representation is symmetric with regards to any permutation of its components, e.g., $T_{a,b}^{\left(m,n\right)}=T_{b,a}^{\left(n,m\right)}$, and $T_{a,b,c}^{\left(l,m,n\right)}=T_{c,a,b}^{\left(n,l,m\right)}=T_{c,b,a}^{\left(n,m,l\right)}$, and so on.
(iv) The transformations can all be done by way of the table method explained in the last section. That is, we do not have to carry out tedious matrix multiplications, but can instead just use Table 1 suitably implemented into code to convert the Cartesians into spherical harmonic components.

At this stage it will prove useful to return to the diagrammatic representation.

**Figure 5:**
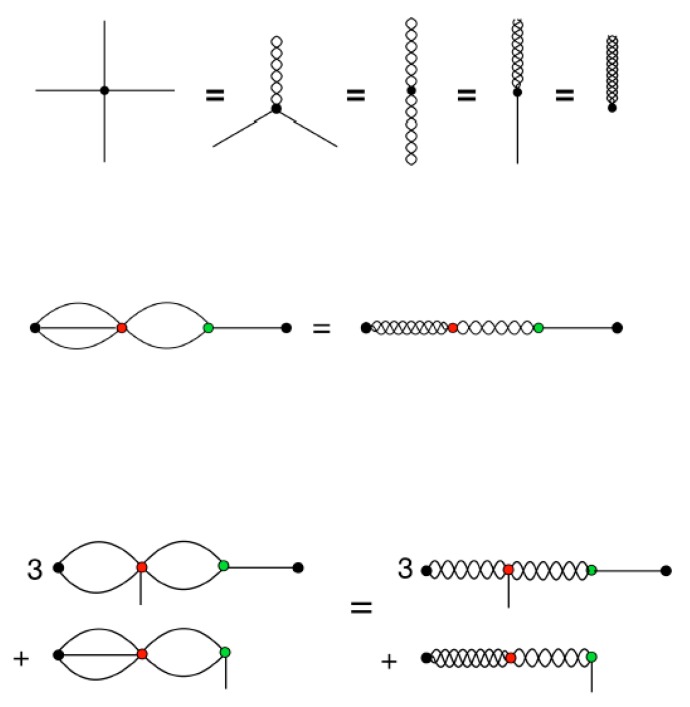
Equivalence of Cartesian and spherical harmonic representations. Top line: Four equivalent ways of representing a rank-4 symmetric traceless tensor. Middle line: two equivalent ways of representing the $\left(M^{i\left(5\right)}\vdots R^{ji\left(3\right)}\right):\left(M^{j\left(3\right)}.R^{ji\left(1\right)}\right)$ tensor product from Figure 1. Bottom: two equivalent ways of representing the gradient of this tensor product. The unbraided spokes represent Cartesian vectors, and the braided spokes are spherical harmonics.

Figure 5’s top illustrates the equivalence between different representations of the tensors in spherical harmonics and Cartesian coordinates. The example given is of a traceless symmetric rank 4 tensor, which can be represented as either $T^{\left(1,1,1,1\right)}$, or $T^{\left(2,1,1\right)}$, or $T^{\left(2,2\right)}$, or $T^{\left(3,1\right)}$, or $T^{\left(4\right)}$, where the Cartesian coordinates are, as usual, represented by spokes, and where the transformation to spherical harmonics is depicted by *braiding* any number of spokes together. Of course, this is just a visual metaphor, but it is intended to convey how the transformation into spherical harmonics intertwines (through taking linear combinations of) multiple Cartesian indices into one spherical harmonic index.

The next line, in Figure 5’s middle, shows how energy term, $\left(M^{i\left(5\right)}\vdots R^{ji\left(3\right)}\right):\left(M^{j\left(3\right)}.R^{ji\left(1\right)}\right)$, depicted in Figure 1, can be converted to spherical harmonics, through performing the braidings $M_{\alpha \beta \gamma \delta \epsilon }^{i\left(5\right)}\to M_{a,b}^{i\left(3,2\right)}$, $M_{\alpha \beta \gamma }^{j\left(3\right)}\to M_{a,b}^{j\left(2,1\right)}$, $R_{\alpha \beta }^{ji\left(1\right)}\to q^{a\left(1\right)}\left(r^{ji}\right)$ and $R_{\alpha \beta \gamma }^{ji\left(3\right)}\to q^{a\left(3\right)}\left(r^{ji}\right)$, and then calculating the contractions according to

(68) $$\left(M^{i\left(5\right)}\vdots R^{ji\left(3\right)}\right):\left(M^{j\left(3\right)}.R^{ji\left(1\right)}\right)=\overset{7}{\underset{a=1}{\sum^{}}}\overset{5}{\underset{b=1}{\sum^{}}}q^{b\left(3\right)}\left(r^{ji}\right)M_{b,a}^{i\left(3,2\right)}\overset{3}{\underset{c=1}{\sum^{}}}M_{a,c}^{j\left(2,1\right)}q^{c\left(1\right)}\left(r^{ji}\right).$$

Furthermore, the final diagrammatic equation, in Figure 5’s bottom, shows how to convert the gradient of the above term into spherical harmonics, which requires the additional braiding $M_{\alpha \beta \gamma \delta \epsilon }^{i\left(5\right)}\to M_{a,\nu ,c}^{i\left(2,1,2\right)}$.

The methods developed here can be used to transform any contraction, and thus, we are now in a position to transform the entire multipole interaction, energies and forces and fields, into spherical harmonics. We begin with the multipole interaction generating formula of Equation (31), which in spherical harmonics is given by

(69) $$G_{ji}^{l}\left(r^{ji}\right)=\underset{d_{i}+d_{c}+d_{j}=l}{\sum^{}}C_{d_{i},d_{c},d_{j}}\overset{2d_{c}+1}{\underset{a=1}{\sum^{}}}\overset{2d_{i}+1}{\underset{b=1}{\sum^{}}}q^{b\left(d_{i}\right)}\left(r^{ji}\right)M_{b,a}^{i\left(d_{i},d_{c}\right)}\overset{2d_{j}+1}{\underset{c=1}{\sum^{}}}M_{a,c}^{j\left(d_{c},d_{j}\right)}q^{c\left(d_{j}\right)}\left(r^{ji}\right).$$

The spherical harmonic analogue to Equation (34), which gives the gradient terms necessary for the force calculations (per Equation (31)) is given by

(70) $$\begin{array}{ll} \frac{\partial }{\partial r_{\gamma }^{j}}G_{ji}^{l}\left(r^{ji}\right)= & \sum_{d_{i}+d_{c}+d_{j}=l}C_{d_{i},d_{c},d_{j}}\sum_{a}^{2d_{c}+1}[d_{i}\sum_{b=1}^{2d_{i}-1}q^{b\left(d_{i}-1\right)}\left(r^{ji}\right)M_{b,\gamma ,a}^{i\left(d_{i}-1,1,d_{c}\right)}\sum_{c=1}^{2d_{j}+1}M_{a,c}^{j\left(d_{c},d_{j}\right)}q^{c\left(d_{j}\right)}\left(r^{ji}\right)\\ & +d_{j}\sum_{c=1}^{2d_{i}+1}q^{c\left(d_{i}\right)}\left(r^{ji}\right)M_{c,a}^{i\left(d_{i},d_{c}\right)}\sum_{b=1}^{2d_{j}-1}M_{a,\gamma ,b}^{j\left(d_{c},1,d_{j}-1\right)}q^{b\left(d_{j}-1\right)}\left(r^{ji}\right)] \end{array}$$

and the spherical harmonic analogue to Equation (38), which gives the derivatives necessary for the multipole fields (per Equation (37)) is given by

(71) $$\frac{\partial }{\partial M^{j\left(n\right)}}G_{ji}^{l}\left(r^{ji}\right)=\mathrm{SYMM}\left[\underset{\underset{d_{c}+d_{j}=n}{d_{i}+d_{j}+d_{c}=l}}{\sum^{}}C_{d_{i},d_{c},d_{j}}\left(\overset{2d_{i}+1}{\underset{b=1}{\sum^{}}}q^{b\left(d_{i}\right)}\left(r^{ji}\right)M_{b,a}^{i\left(d_{i},d_{c}\right)}\right)q^{c\left(d_{j}\right)}\left(r^{ji}\right)\right].$$

This last expression needs some explanation. Each term in the sum has a tensor representation of type $T_{a,c}^{\left(d_{c},d_{j}\right)}$, but given that the sum is over $d_{c},d_{j}$, the quantity in square brackets will result in a sum in different split component representations, e.g., for rank 4, the sum will have the form $S^{\left(4\right)}=c_{4}T^{\left(4\right)}+c_{3,1}T^{\left(3,1\right)}+c_{2,2}T^{\left(2,2\right)}$, where the representations do not in general refer to symmetric tensors. However, once the full sum has been evaluated, it can be symmetrised by converting the result back into Cartesians, before averaging over all permutations of the Cartesian indices.

The electrostatic potential at r, from a multipole expansion at the origin can be found from the above by taking the rank 0 multipole derivative and then using Equation (37) to obtain

(72) $$\phi \left(r\right)=\overset{\infty }{\underset{l=0}{\sum^{}}}B_{l}\left(r\right)\overset{2l+1}{\underset{a=1}{\sum^{}}}q^{a\left(l\right)}\left(r\right)M_{a}^{\left(l\right)}=\overset{\infty }{\underset{l=0}{\sum^{}}}B_{l}\left(r\right){\left\langle M^{\left(l\right)},R^{\left(l\right)}\right\rangle }_{t}$$

where the last term can be derived from the first, or obtained from taking the rank 0 multipole derivative of Equation (38), and where we note that it reduces to Equation (22) for the kernel $B_{0}\left(r\right)=1/r.$

Finally, the spherical harmonic analogue for Equation (42), giving the total torque on a multipole site is given by

(73) $$t_{\alpha }=-\overset{\infty }{\underset{n=1}{\sum^{}}}n\overset{2n-1}{\underset{i=1}{\sum^{}}}\underset{\beta \gamma }{\sum^{}}\epsilon_{\alpha \beta \gamma }M_{i,\beta }^{\left(n-1,1\right)}\phi_{i,\gamma }^{\left(n-1,1\right)}$$

Equations (69)–(73) then comprise our final expressions for the multipole interaction in spherical harmonics, in a form suitable for implementation into the Ewald sum. Here, we should admit that we have not given spherical harmonic equivalents for rotation of the multipoles, or for calculation of the angular derivatives (Equation (39)). We currently prefer to keep these in Cartesians for simplicity, but given that both these calculations can be performed outside the main *ij* particle loop, there is no significant computational cost to their calculation.

### 3.2. Implementation

It may be a cause for worry that implementing the expressions in the last section is technically very difficult, or computationally costly; however, neither of these things is true.

As far as the implementation goes, it is true that, due to the amount of ‘book-keeping’ required, implementing the multipole interaction in either Cartesians or spherical harmonics is a moderately difficult coding task, but implementing Equations (69)–(73), does not need to be any more difficult than implementing the same sums in Cartesians.

As for computational cost, note that, contrary to a perennial myth, implementation of the multipole interaction in spherical harmonics does not require the calculation of any expensive trigonometric functions. All of the necessary coordinate transformations can be done using Table 1, suitably implemented into code, obviating the need for any explicit matrix multiplication or calculation of trigonometric functions (see Section 4.7). It is true that the spherical harmonic transformations may need to be evaluated afresh each step of a simulation, but the required transformations can be done exclusively outside the main *ij* particle loop, which almost always takes up the vast majority of time in a calculation. These transformations can be done in a time that’s linear with the number of particles, and which, in any case require no operations more complex than the multiplication of real numbers. Furthermore, the final expressions in spherical harmonics require fewer operations than their Cartesian equivalents to calculate, thus providing an overall saving in computational time.

Implementation is greatly aided by the use of tests to verify the results at each stage.

We begin with the energies, where numerical differentiation can be used to check that the multipole interactions are giving the right energies for each rank.

Suppose we are confident that the multipole interactions are accurate to rank *n*. Then, it can be checked that the rank *n* + 1 multipoles are also giving the right results by comparing the analytic energies for rank *n* + 1 multipoles against numerical results found from numerical differentiation of the rank n multipoles.

One way of doing this is, given rank-*n* multipole $M^{\left(n\right)}$, we construct a fictitious diatom of bond-length Δr, in which the first site holds a multipole $M^{\left(n\right)}/\Delta r$, and the second site holds a multipole $-M^{\left(n\right)}/\Delta r$. Then, as Δr→0, a multipole expansion of the diatom as a whole will give a pure rank *n* + 1 multipole moment. We can now check to see how the energy of this diatom in the field of other rank ≤ *n* multipoles compares to the energy of the system when replacing it by an analytic multipole of rank *n* + 1, where the analytic multipole is assigned the rank *n* + 1 multipole moments of the diatom.

If Δr is made small enough, it should be possible to obtain exact agreement up to numerical precision. Furthermore, in this way, it is possible to boot-strap our way to checking multipoles of arbitrary rank. We begin with charges, which can be added together to make numerical dipoles. Furthermore, once the analytic dipoles are confirmed, pairs of analytic dipoles can be added to together to make numerical quadrupoles, and so on.

We have implemented our expressions for the energies, fields, forces and torques in spherical harmonics into an Ewald sum code, going up to rank 3 in the multipole expansion. The analytic forces’ torques and angular derivatives were checked by comparison to numerical derivatives.

The reciprocal space and self-interaction parts of the Ewald sum are given in Section 4.7, and given that both these terms involve simple to convert inner products over the multipoles, it is absolutely straightforward to convert the Cartesian multipole form of these expressions as given by Smith [1] into their spherical harmonic equivalents.

We tested our code on a system of 32 molecules each containing 26 nuclei and interacting under periodic boundary conditions, and found about a 22% speed-up upon converting the full Ewald sum to spherical harmonics for multipoles up to rank 3, where the Cartesian form had already been heavily optimised to remove all obviously redundant operations. This is a not insignificant saving, and the difference would only be expected to grow with increasing rank.

We have made a copy of our code available on the Internet [21]. It includes all the gradient tests mentioned in this section, and also includes the aforementioned multipole consistency test, in which numerical rank *n* + 1 multipoles are created from displacing rank n multipoles.

### 3.3. Scaling

The implementation of site multipole expansions does not alter the fundamental scaling with respect to the number of particles over that of a calculation involving just point charges, but there is a scaling with respect to the maximum multipole rank used in the expansion, and it is to this we now turn.

To obtain an estimate of this scaling, we will try enumerating the number of multiplications involved in calculating the $G_{ji}^{l}\left(r\right)$ functions from Equation (31) up to a given rank.

Recall that the inner product of two rank-n tensors requires a minimum of (n+1)(n+2)/2 multiplications in Cartesians (accounting for permutation symmetry), and 2*n* + 1 multiplications in spherical harmonics. Then, the inner product of Equation (31), which has $d_{i}$ left-bracket contractions, $d_{j}$ right-bracket contractions, and $d_{c}$ between-bracket contractions requires

(74) $$X_{d_{i},d_{c},d_{j}}^{cart}=\left(\left(d_{i}+1\right)\left(d_{i}+2\right)+\left(d_{c}+1\right)\left(d_{c}+2\right)+\left(d_{j}+1\right)\left(d_{j}+2\right)\right)/2-\delta_{d_{i},0}-\delta_{d_{j},0}$$

multiplications in Cartesians and

(75) $$X_{d_{i},d_{c},d_{j}}^{SH}=\left(2d_{i}+1\right)+\left(2d_{c}+1\right)+\left(2d_{j}+1\right)-\delta_{d_{i},0}-\delta_{d_{j},0}$$

multiplications in spherical harmonics, where the $-\delta_{d_{i},0}-\delta_{d_{j},0}$ terms in the above two expressions arise from the fact that if $d_{i}=0$, then the corresponding $R^{ji}$ tensor is equal to unity and no multiplication is required (and similarly for $d_{j}=0$).

To obtain the total number of multiplications involved in calculating the $G_{ji}^{l}\left(r\right)$ functions for multipoles up to a given rank, we wrote a simple code to sum the values

(76) $$X\left(n\right)=\underset{l}{\sum^{}}\underset{\underset{d_{i}+d_{c},d_{j}+d_{c}\leq n}{d_{i}+d_{j}+d_{c}=l}}{\sum^{}}X_{d_{i},d_{c},d_{j}},$$

where X(n) is the total number of multiplications, and where we are only summing over terms with multipole ranks $d_{i}+d_{c},d_{j}+d_{c}\leq n$.

Finally, to obtain the scaling, we fit the curves ΔX(n)=X(n)−X(0) up to a maximum rank of n=8, with the form $\Delta X=an^{s}$, where a,s are fitting coefficients, with s being the scaling power.

The result of this exercise was that we found $\Delta X^{cart}\left(n\right)=3.5n^{3.8}$ for Cartesians and $\Delta X^{SH}\left(n\right)=5.8n^{3.3}$ in spherical harmonics. Thus, the spherical harmonics are expected to have better scaling than the Cartesian case (s= 3.3 for spherical harmonics vs. 3.8 for Cartesians.)

It is hard to imagine many users would want to go beyond *n* = 8, but repeating the above in the range *n* = 1…16 gives a scaling of s= 3.6 for spherical harmonics vs. 4.3 for Cartesians.

This analysis is admittedly quite crude. It does not consider the cost of calculating the forces, the fields, or the cost of array look-ups. It also ignores the fact that at least some of the inner products occur more than once in the calculation of the $G_{ji}^{l}\left(r\right)$ functions, and so only need to be evaluated once and stored for later use. In light of this, it is worth discussing how our actual implementations perform.

To this end, we ran a 32-molecule test case with each molecule having 26 atoms, for both periodic and non-periodic boundary conditions, and for both spherical harmonics and Cartesian implementations of the multipole interactions, where the periodic simulations employed a full Ewald sum.

We recognise that some groups will be interested in calculations using much larger system sizes, but our code is optimised for crystal-structure prediction using relatively small numbers of molecules, as it often makes sense to look for crystal structures with relatively small simulation cells. Here, we also mention that, unlike many codes, our code allows for arbitrary cut-off radii, [22] where the cut-off sphere is allowed to be larger than can fit in the simulation cell, which means that we can converge energies for even small unit cells, and in this case we employed a real space cut-off of 16 Å.

For the periodic calculation we obtained scalings of s = 3.7 for spherical harmonics, and s = 4.5 for Cartesians, and for the non-periodic calculation we obtained *s* = 2.7 for spherical harmonics and s = 3.3 for Cartesians; results that are not too dissimilar from our relatively crude theoretical predictions.

## 4. Materials and Methods

We discuss in this section, by way of technical sub-sections, the underlying intellectual and mathematical infrastructure underpinning the above-discussed novel contributions of the present work outlined in Section 2 and discussed further in Section 3, before summarising and concluding the article in Section 5 below.

### 4.1. Some Mathematical Properties of Cartesian Tensors and Notation

Let v be a Cartesian vector in R^3^, which has Cartesian components $v.\hat{\alpha }=v_{\alpha }$, where α is one of x, y or z, and $\hat{\alpha }$ is a unit vector in the α direction. If x, y, z is an orthogonal axis set, then $\hat{\alpha }.\hat{\beta }=\delta_{\alpha ,\beta }$, where $\delta_{\alpha ,\beta }=1$ if α=β, and $\delta_{\alpha ,\beta }=0$ otherwise. Thus, we can write $v=\sum_{\alpha }v_{\alpha }\hat{\alpha }$, where the sum is over the three directional indices, x, y and z.

Now suppose we also have a reference set of axes, given by ${\hat{x}}^{ref}$, ${\hat{y}}^{ref}$ and ${\hat{z}}^{ref}$, where ${\hat{\alpha }}^{ref}.{\hat{\beta }}^{ref}=\delta_{\alpha ,\beta }$, then $O_{\alpha \beta }=\hat{\alpha }.{\hat{\beta }}^{ref}$ is the orthogonal 3 × 3 rotation matrix, which takes the reference-frame components to the components in the laboratory frame via

(77) $$v_{\alpha }=\underset{\beta }{\sum^{}}O_{\alpha \beta }v_{\beta }^{ref}$$

Now, let $T^{\left(n\right)}=\underset{\alpha \beta ..}{\sum^{}}T_{\alpha \beta \gamma ..}^{\left(n\right)}\hat{\alpha }\hat{\beta }\hat{\gamma }..$ be a *tensor* of rank n, which has Cartesian components $T_{\alpha \beta \gamma ..}^{\left(n\right)}$, where the number of indices is equal to its rank, and where the tensor components transform according to

(78) $$T_{\alpha \beta \gamma ..}^{\left(n\right)}=\underset{\alpha^{\prime }\beta^{\prime }\gamma^{\prime }..}{\sum^{}}O_{\alpha \alpha^{\prime }}O_{\beta \beta^{\prime }}O_{\gamma \gamma^{\prime }}..T_{\alpha^{\prime }\beta^{\prime }\gamma^{\prime }..}^{ref\left(n\right)}=\underset{\alpha^{\prime }\beta^{\prime }\gamma^{\prime }..}{\sum^{}}O_{\alpha \beta \gamma ..,\alpha^{\prime }\beta^{\prime }\gamma^{\prime }}^{\left(n\right)}T_{\alpha^{\prime }\beta^{\prime }\gamma^{\prime }..}^{ref\left(n\right)},$$

where there are n occurrences of the rotation matrix in the above, and where we have defined the orthogonal tensor rotation *matrix* $O^{\left(n\right)}$, with components

(79) $$O_{\alpha \beta \gamma ..,\alpha^{\prime }\beta^{\prime }\gamma^{\prime }}^{\left(n\right)}=O_{\alpha \alpha^{\prime }}O_{\beta \beta^{\prime }}O_{\gamma \gamma^{\prime }}..,$$

We will generally use the superscript notation (n) to indicate the rank of each tensor, except in a small number of cases where the rank can be inferred from counting its indices. To distinguish different tensors of the same kind and rank, we will often also use the superscript to give labels to the tensors, e.g., if we have two rank-n tensors we wish to label a and b, then we will use the notation $T^{a\left(n\right)}$ and $T^{b\left(n\right)}$, which have components $T_{\alpha \beta \gamma ..}^{a\left(n\right)}$ and $T_{\alpha \beta \gamma ..}^{b\left(n\right)}$.

Almost all of the tensors used in this work are symmetric with respect to the permutation of their indices, e.g., $T_{\alpha \beta \gamma }=T_{\alpha \gamma \beta }=T_{\beta \gamma \alpha }=T_{\beta \alpha \gamma }=T_{\gamma \alpha \beta }=T_{\gamma \beta \alpha }$, and we will refer to such tensors as *symmetric*.

We now turn to a discussion of tensor contractions. We will only give a brief overview, but we note that Applequist has written extensively on this topic, and the interested reader should consult his work [15].

Suppose that $A^{\left(i\right)}$ and $B^{\left(j\right)}$ are two such symmetric tensors. We introduce the notation A:n:B to indicate a *contraction* over n indices of two such tensors, e.g.,

(80) $$G_{\gamma \delta \epsilon }^{\left(3\right)}={\left(A^{\left(3\right)}.2.B^{\left(4\right)}\right)}_{\gamma \delta \epsilon }=\underset{\alpha \beta }{\sum^{}}A_{\alpha \beta \gamma }^{\left(3\right)}B_{\alpha \beta \delta \epsilon }^{\left(4\right)},$$

is a contraction over 2 indices, and

(81) $$G_{\delta }^{\left(1\right)}={\left(A^{\left(3\right)}.3.B^{\left(4\right)}\right)}_{\delta }=\underset{\alpha \beta \gamma }{\sum^{}}A_{\alpha \beta \gamma }^{\left(3\right)}B_{\alpha \beta \gamma \delta }^{\left(4\right)},$$

is a contraction over 3 indices.

Furthermore, in general, the contraction $A^{\left(i\right)}.n.B^{\left(j\right)}$ results in a symmetric tensor of rank *i* + *j* − 2*n*.

For small numbers of contractions, we can use the alternative notation that a contraction is indicated by vertical dots, where the number of dots is equal to the degree of the contraction, e.g., $A^{\left(i\right)}.B^{\left(j\right)}=A^{\left(i\right)}.1.B^{\left(j\right)}$, $A^{\left(i\right)}:B^{\left(j\right)}=A^{\left(i\right)}.2.B^{\left(j\right)}$, and $A^{\left(i\right)}\vdots B^{\left(j\right)}=A^{\left(i\right)}.3.B^{\left(j\right)}$.

Now consider the contraction $s=A^{\left(n\right)}.n.B^{\left(n\right)}$, where $A^{\left(n\right)}$, and $B^{\left(n\right)}$ are traceless rank (*n*) tensors, and the result, s, is a scalar. It is easy to show that this contraction forms an inner product on the vector space of all rank-d tensors. Thus, we will use the notation

(82) $${\left\langle A^{\left(n\right)},B^{\left(n\right)}\right\rangle }_{t}=A^{\left(n\right)}.n.B^{\left(n\right)}=\underset{\alpha \beta \gamma ..}{\sum^{}}A_{\alpha \beta \gamma ..}^{\left(n\right)}B_{\alpha \beta \gamma ..}^{\left(n\right)}.$$

To prove that the above is a genuine inner product, we first use the fact that symmetric rank-n tensors form a vector space, that is, the addition of any two rank-*n* symmetric tensors results in another rank-*n* symmetric tensor, and the multiplication of any symmetric rank-*n* tensor by a scalar also results in another rank-n symmetric tensor. Then, we can show that on this vector space, (i) ${\left\langle A^{\left(n\right)},B^{\left(n\right)}\right\rangle }_{t}$ is always a scalar. (ii) for any scalar, s: $s{\left\langle A^{\left(n\right)},B^{\left(n\right)}\right\rangle }_{t}={\left\langle sA^{\left(n\right)},B^{\left(n\right)}\right\rangle }_{t}$ (linearity), (iii) ${\left\langle A^{\left(n\right)},B^{\left(n\right)}\right\rangle }_{t}={\left\langle B^{\left(n\right)},A^{\left(n\right)}\right\rangle }_{t}$ (symmetry), and (iv) ${\left\langle A^{\left(n\right)},A^{\left(n\right)}\right\rangle }_{t}\geq 0$. (positive defiteness). Which are the four conditions required for ${\left\langle A^{\left(n\right)},B^{\left(n\right)}\right\rangle }_{t}$ to be an inner product.

It remains to be shown that the inner product is the same in any axis frame, such that it operates on the tensors, and not just their components.

Firstly, working in the lab-axis frame, if a,b,c,d are any rank 1 Cartesian vectors, then ab:cd=(a.c)(b.d). From which it follows that if we have axes defined by orthogonal unit vectors $\hat{\alpha }$ and ${\hat{\alpha }}^{\prime }$, where one is rotated with respected to the other, then $\hat{\alpha }\hat{\beta }:{\hat{\gamma }}^{\prime }{\hat{\delta }}^{\prime }=A_{\alpha \gamma }A_{\beta \delta }$, where $A_{\alpha \gamma }=\alpha .{\hat{\gamma }}^{\prime }$ are the components of the rotation matrix which transforms between the two frames.

Now, suppose we expand out the inner product of two rank 2 tensors $C^{\left(2\right)}$ and $D^{\left(2\right)}$, where the latter’s components are calculated in the ${\hat{\alpha }}^{\prime }$ frame, then, writing $D_{\alpha \beta }^{\prime }$ for the components in this frame we have

(83) $$\underset{\alpha \beta }{\sum^{}}C_{\alpha \beta }\hat{\alpha }\hat{\beta }:\underset{\gamma \delta }{\sum^{}}D_{\gamma \delta }^{\prime }{\hat{\gamma }}^{\prime }{\hat{\delta }}^{\prime }=\underset{\alpha \beta }{\sum^{}}C_{\alpha \beta }\underset{\gamma \delta }{\sum^{}}A_{\alpha \gamma }A_{\beta \delta }D_{\gamma \delta }^{\prime }=\underset{\alpha \beta }{\sum^{}}C_{\alpha \beta }D_{\alpha \beta }$$

which is the same as if both tensors components were calculated in the same frame. Thus, ${\left\langle C^{\left(n\right)},D^{\left(n\right)}\right\rangle }_{t}$ is the same no matter the frame each tensor’s components are calculated in, and the same goes for all tensor inner products in general.

Finally, it is useful to define the tensors

(84) $$R^{\left(n\right)}=rrrr..,$$

with components $R_{\alpha \beta \gamma ..}^{\left(n\right)}=r_{\alpha }r_{\beta }r_{\gamma }r_{\delta }..$, where the number of rs is equal to its rank. It can be readily seen that $R^{\left(m\right)}R^{\left(n\right)}=R^{\left(m+n\right)}$, which implies that $R^{\left(0\right)}=1$.

Similarly, we define the tensors

(85) $$\nabla^{\left(n\right)}=\nabla \nabla \nabla \nabla ..,$$

where $\nabla_{\alpha }=\partial /\partial r_{\alpha }$, and $\nabla_{\alpha \beta \gamma ..}^{\left(n\right)}=\partial^{n}/\partial r_{\alpha }\partial r_{\beta }\partial r_{\gamma }..\;Furthermore,$ again, we have that $\nabla^{\left(m\right)}\nabla^{\left(n\right)}=\nabla^{\left(m+n\right)}$, which implies that $\nabla^{\left(0\right)}=1$.

### 4.2. The $G_{ji}^{l}\left(r\right)$ Functions up to Rank 3

The following lists the $G_{ji}^{l}\left(r\right)$ functions defined from Equation (31) (and surrounding text) up to multipoles of rank 3. These formulae agree with those derived by Smith, [1] who calculated terms up to rank 3, except that here we list only those terms which occur for traceless multipole tensors.

(86) $$G_{ji}^{0}\left(r^{ji}\right)=\left(M^{i\left(0\right)}\right)\left(M^{j\left(0\right)}\right)$$

(87) $$\begin{array}{c} G_{ji}^{1}\left(r^{ji}\right)=\left(M^{i\left(1\right)}.R^{ji\left(1\right)}\right)\left(M^{j\left(0\right)}\right)-\left(M^{j\left(1\right)}.R^{ji\left(1\right)}\right)\left(M^{i\left(0\right)}\right)\\ +\left(M^{i\left(1\right)}\right).\left(M^{j\left(1\right)}\right) \end{array}$$

(88) $$\begin{array}{c} G_{ji}^{2}\left(r^{ji}\right)=-\left(M^{i\left(1\right)}.R^{ji\left(1\right)}\right)\left(M^{j\left(1\right)}.R^{ji\left(1\right)}\right)\\ +2\left(M^{i\left(2\right)}.R^{ji\left(1\right)}\right).\left(M^{j\left(1\right)}\right)-2\left(M^{j\left(2\right)}.R^{ji\left(1\right)}\right).\left(M^{i\left(1\right)}\right)\\ +\left(M^{i\left(2\right)}:R^{ji\left(2\right)}\right)\left(M^{j\left(0\right)}\right)+\left(M^{j\left(2\right)}:R^{ji\left(2\right)}\right)\left(M^{i\left(0\right)}\right)\\ +2\left(M^{i\left(2\right)}\right):\left(M^{j\left(2\right)}\right) \end{array}$$

(89) $$\begin{array}{c} G_{ji}^{3}\left(r^{ji}\right)=-4\left(M^{i\left(2\right)}.R^{ji\left(1\right)}\right).\left(M^{j\left(2\right)}.R^{ji\left(1\right)}\right)\\ -\left(M^{i\left(2\right)}:R^{ji\left(2\right)}\right)\left(M^{j\left(1\right)}.R^{ji\left(1\right)}\right)+\left(M^{j\left(2\right)}:R^{ji\left(2\right)}\right)\left(M^{i\left(1\right)}.R^{ji\left(1\right)}\right)\\ +\left(M^{i\left(3\right)}\vdots R^{ji\left(3\right)}\right)M^{j\left(0\right)}-\left(M^{j\left(3\right)}\vdots R^{ji\left(3\right)}\right)M^{i\left(0\right)}\\ +3\left(M^{i\left(1\right)}\right).\left(M^{j\left(3\right)}:R^{ji\left(2\right)}\right)+3\left(M^{j\left(1\right)}\right).\left(M^{i\left(3\right)}:R^{ji\left(2\right)}\right)\\ -6\left(M^{i\left(2\right)}\right):\left(M^{j\left(3\right)}.R^{ji\left(1\right)}\right)+6\left(M^{j\left(2\right)}\right):\left(M^{i\left(3\right)}.R^{ji\left(1\right)}\right)\\ +6\left(M^{i\left(3\right)}\right)\vdots \left(M^{j\left(3\right)}\right) \end{array}$$

(90) $$\begin{array}{c} G_{ji}^{4}\left(r^{ji}\right)=\left(M^{i\left(2\right)}:R^{ji\left(2\right)}\right)\left(M^{j\left(2\right)}:R^{ji\left(2\right)}\right)\\ -\left(M^{i\left(3\right)}\vdots R^{ji\left(3\right)}\right)\left(M^{j\left(1\right)}.R^{ji\left(1\right)}\right)-\left(M^{j\left(3\right)}\vdots R^{ji\left(3\right)}\right)\left(M^{i\left(1\right)}.R^{ji\left(1\right)}\right)\\ -6\left(M^{i\left(3\right)}:R^{ji\left(2\right)}\right).\left(M^{j\left(2\right)}.R^{ji\left(1\right)}\right)+6\left(M^{j\left(3\right)}:R^{ji\left(2\right)}\right).\left(M^{i\left(2\right)}.R^{ji\left(1\right)}\right)\\ -18\left(M^{i\left(3\right)}.R^{ji\left(1\right)}\right):\left(M^{j\left(3\right)}.R^{ji\left(1\right)}\right) \end{array}$$

(91) $$\begin{array}{c} G_{ji}^{5}\left(r^{ji}\right)=\left(M^{i\left(3\right)}\vdots R^{ji\left(3\right)}\right)\left(M^{j\left(2\right)}:R^{ji\left(2\right)}\right)-\left(M^{j\left(3\right)}\vdots R^{ji\left(3\right)}\right)\left(M^{i\left(2\right)}:R^{ji\left(2\right)}\right)\\ +9\left(M^{i\left(3\right)}:R^{ji\left(2\right)}\right).\left(M^{j\left(3\right)}:R^{ji\left(2\right)}\right) \end{array}$$

(92) $$G_{ji}^{6}\left(r^{ji}\right)=-\left(M^{i\left(3\right)}\vdots R^{ji\left(3\right)}\right)\left(M^{j\left(3\right)}\vdots R^{ji\left(3\right)}\right)$$

### 4.3. The $\partial G_{ji}^{l}\left(r^{ji}\right)/\partial r^{j}$ Functions up to Rank 3

The following lists the $\partial G_{ji}^{l}\left(r^{ji}\right)/\partial r^{j}$ functions given by Equation (34), up to rank 3 in the multipoles. These formulae agree with those derived by Smith, [1] who calculated terms up to rank 3, except that here we list only those terms which occur for traceless multipole tensors.

(93) $$\begin{array}{c} \partial G_{ji}^{0}\left(r^{ji}\right)/\partial r^{j}=0 \end{array}$$

(94) $$\begin{array}{c} \partial G_{ji}^{1}\left(r^{ji}\right)/\partial r^{j}=\left(M^{i\left(1\right)}\right)\left(M^{j\left(0\right)}\right)-\left(M^{j\left(1\right)}\right)\left(M^{i\left(0\right)}\right) \end{array}$$

(95) $$\begin{array}{c} \partial G_{ji}^{2}\left(r^{ji}\right)/\partial r^{j}=-\left(M^{i\left(1\right)}\right)\left(M^{j\left(1\right)}.R^{ji\left(1\right)}\right)-\left(M^{i\left(1\right)}.R^{ji\left(1\right)}\right)\left(M^{j\left(1\right)}\right)\\ +2\left(M^{i\left(2\right)}\right).\left(M^{j\left(1\right)}\right)-2\left(M^{j\left(2\right)}\right).\left(M^{i\left(1\right)}\right)\\ +2\left(M^{i\left(2\right)}.R^{ji\left(1\right)}\right)\left(M^{j\left(0\right)}\right)+2\left(M^{j\left(2\right)}.R^{ji\left(1\right)}\right)\left(M^{i\left(0\right)}\right) \end{array}$$

(96) $$\begin{array}{c} \partial G_{ji}^{3}\left(r^{ji}\right)/\partial r^{j}=-4\left(M^{i\left(2\right)}\right).\left(M^{j\left(2\right)}.R^{ji\left(1\right)}\right)-4\left(M^{i\left(2\right)}.R^{ji\left(1\right)}\right).\left(M^{j\left(2\right)}\right)\\ -2\left(M^{i\left(2\right)}.R^{ji\left(1\right)}\right)\left(M^{j\left(1\right)}.R^{ji\left(1\right)}\right)-\left(M^{i\left(2\right)}:R^{ji\left(2\right)}\right)\left(M^{j\left(1\right)}\right)\\ +2\left(M^{j\left(2\right)}.R^{ji\left(1\right)}\right)\left(M^{i\left(1\right)}.R^{ji\left(1\right)}\right)+\left(M^{j\left(2\right)}:R^{ji\left(2\right)}\right)\left(M^{i\left(1\right)}\right)\\ +3\left(M^{i\left(3\right)}:R^{ji\left(2\right)}\right)M^{j\left(0\right)}-3\left(M^{j\left(3\right)}:R^{ji\left(2\right)}\right)M^{i\left(0\right)}\\ +6\left(M^{i\left(1\right)}\right).\left(M^{j\left(3\right)}.R^{ji\left(1\right)}\right)+6\left(M^{j\left(1\right)}\right).\left(M^{i\left(3\right)}.R^{ji\left(1\right)}\right)\\ -6\left(M^{i\left(2\right)}\right):\left(M^{j\left(3\right)}\right)+6\left(M^{j\left(2\right)}\right):\left(M^{i\left(3\right)}\right) \end{array}$$

(97) $$\begin{array}{c} \partial G_{ji}^{4}\left(r^{ji}\right)/\partial r^{j}=\\ 2\left(M^{i\left(2\right)}.R^{ji\left(1\right)}\right)\left(M^{j\left(2\right)}:R^{ji\left(2\right)}\right)+2\left(M^{i\left(2\right)}:R^{ji\left(2\right)}\right)\left(M^{j\left(2\right)}.R^{ji\left(1\right)}\right)\\ -3\left(M^{i\left(3\right)}:R^{ji\left(2\right)}\right)\left(M^{j\left(1\right)}.R^{ji\left(1\right)}\right)-\left(M^{i\left(3\right)}\vdots R^{ji\left(3\right)}\right)\left(M^{j\left(1\right)}\right)\\ -3\left(M^{j\left(3\right)}:R^{ji\left(2\right)}\right)\left(M^{i\left(1\right)}.R^{ji\left(1\right)}\right)-\left(M^{j\left(3\right)}\vdots R^{ji\left(3\right)}\right)\left(M^{i\left(1\right)}\right)\\ -12\left(M^{i\left(3\right)}.R^{ji\left(1\right)}\right).\left(M^{j\left(2\right)}.R^{ji\left(1\right)}\right)-6\left(M^{i\left(3\right)}:R^{ji\left(2\right)}\right).\left(M^{j\left(2\right)}\right)\\ +12\left(M^{j\left(3\right)}.R^{ji\left(1\right)}\right).\left(M^{i\left(2\right)}.R^{ji\left(1\right)}\right)+6\left(M^{j\left(3\right)}:R^{ji\left(2\right)}\right).\left(M^{i\left(2\right)}\right)\\ -18\left(M^{i\left(3\right)}\right):\left(M^{j\left(3\right)}.R^{ji\left(1\right)}\right)-18\left(M^{i\left(3\right)}.R^{ji\left(1\right)}\right):\left(M^{j\left(3\right)}\right) \end{array}$$

(98) $$\begin{array}{c} \partial G_{ji}^{5}\left(r^{ji}\right)/\partial r^{j}=\\ 3\left(M^{i\left(3\right)}:R^{ji\left(2\right)}\right)\left(M^{j\left(2\right)}:R^{ji\left(2\right)}\right)+2\left(M^{i\left(3\right)}\vdots R^{ji\left(3\right)}\right)\left(M^{j\left(2\right)}.R^{ji\left(1\right)}\right)\\ -3\left(M^{j\left(3\right)}:R^{ji\left(2\right)}\right)\left(M^{i\left(2\right)}:R^{ji\left(2\right)}\right)-2\left(M^{j\left(3\right)}\vdots R^{ji\left(3\right)}\right)\left(M^{i\left(2\right)}.R^{ji\left(1\right)}\right)\\ +18\left(M^{i\left(3\right)}.R^{ji\left(1\right)}\right).\left(M^{j\left(3\right)}:R^{ji\left(2\right)}\right)+18\left(M^{i\left(3\right)}:R^{ji\left(2\right)}\right).\left(M^{j\left(3\right)}.R^{ji\left(1\right)}\right) \end{array}$$

(99) $$\begin{array}{c} \partial G_{ji}^{6}\left(r^{ji}\right)/\partial r^{j}=\\ -3\left(M^{i\left(3\right)}:R^{ji\left(2\right)}\right)\left(M^{j\left(3\right)}\vdots R^{ji\left(3\right)}\right)-3\left(M^{i\left(3\right)}\vdots R^{ji\left(3\right)}\right)\left(M^{j\left(3\right)}:R^{ji\left(2\right)}\right) \end{array}$$

### 4.4. The General Decomposition for Homogeneous Polynomials

This sub-section introduces a standard decomposition for polynomials into mutually orthogonal rotationally invariant subspaces. The results will be used below in Section 4.5, in which we explain how to convert between the tensor and spherical inner products.

A result from the theory of spherical harmonics (e.g., see chapter 2 of ref. [23]): Any degree n homogeneous polynomial, $p^{\left(n\right)}\left(r\right)$, has a unique decomposition given by

(100) $$p^{\left(n\right)}\left(r\right)=\overset{\left\lfloor n/2\right\rfloor }{\underset{m=0}{\sum^{}}}r^{2m}h^{m\left(n-2m\right)}\left(r\right)$$

where $h^{m\left(n-2m\right)}\left(r\right)$ is the *m*th harmonic polynomial, and where, as usual, the degree of the polynomial is placed in brackets, so that $h^{m\left(n-2m\right)}\left(r\right)$ describes a polynomial of degree *n*−2*m*. Furthermore, where, the ⌊n/2⌋ in Equation (77) is the smallest integer less than or equal to n/2, e.g., ⌊8/2⌋=4 and ⌊11/2⌋=5.

The decomposition of Equation (100) is *unique*, because each term in the sum resides in a subspace which is mutually orthogonal under the spherical inner product.

To show this, use the result from the theory of spherical harmonics (e.g., chapter 2 of ref. [23]) that for any two harmonic polynomials of degree m and n, the spherical inner product ${\left\langle h^{a\left(m\right)},h^{b\left(n\right)}\right\rangle }_{s}$ is zero, unless m=n. Thus, if $p^{m\left(n\right)}\left(r\right)=r^{2m}h^{m\left(n-2m\right)}\left(r\right)$, and $p^{m^{\prime }\left(n\right)}\left(r\right)$ $=r^{2m^{\prime }}h^{m^{\prime }\left(n-2m^{\prime }\right)}\left(r\right)$, then the spherical inner product, $\langle p^{m\left(n\right)},p^{m^{\prime }\left(n\right)}\rangle_{s}={\left\langle h^{m\left(n-2m\right)},h^{m^{\prime }\left(n-2m^{\prime }\right)}\right\rangle }_{s}$ is zero unless $m=m^{\prime }$.

The decomposition of Equation (100) is also *complete*, because the dimension of the mth subspace is 2(n−2m)+1, and summing over all subspaces gives a dimension of (n+1)(n+2)/2 (which can be proved by induction), which is the full dimension of the vector space of degree n homogeneous polynomials. (For example, if n=7, then 2n+1=15, and the total dimension is 15+11+7+3=36=(7+1)(7+2)/2.)

Now consider a polynomial in the *m*th subspace, and so of the form $p^{\left(n\right)}\left(r\right)=r^{2m}h^{\left(n-2m\right)}\left(r\right)$, and what happens when that polynomial is rotated.

We can rotate the polynomial through calculating $p^{\left(n\right)}\left(Or\right)$, where O is an orthogonal rotation matrix, and where the result of which is $p^{\left(n\right)}\left(Or\right)=r^{2m}h^{\left(n-2m\right)}\left(Or\right)$. However, given that the rotation of any harmonic polynomial is still a harmonic polynomial of the same degree, we have that $p^{\left(n\right)}\left(Or\right)$ must still belong to the same subspace as $p^{\left(n\right)}\left(r\right)$. Thus, any polynomial which belongs to the *m*th subspace is guaranteed to remain in that subspace after any rotation.

Furthermore, it can be shown (e.g., chapter 2 of ref. [23]) that each subspace is *irreducible*, in the sense that it cannot be further divided into rotationally invariant orthogonal subspaces. (Spherical harmonic polynomials of degree *n* form a 2*n* + 1 dimensional basis for the irreducible representation of SO(3), the group of all rotations in three dimensions. See also, for example, chapter 8 in ref. [24]).

We can also define associated projection operators, ${\hat{d}}_{m}$, such that the *m*th projection operator projects a homogeneous polynomial into the *m*th subspace, according to^.^

(101) $${\hat{d}}_{m}p^{\left(n\right)}\left(r\right)=r^{2m}h^{m\left(n-2m\right)}\left(r\right)$$

It was shown in Equation (44) that a degree-n polynomial can be written in the form

(102) $$p^{\left(n\right)}\left(r\right)=\underset{\alpha \beta \gamma ..}{\sum^{}}P_{\alpha \beta \gamma ..}^{\left(n\right)}R_{\alpha \beta \gamma ..}^{\left(n\right)}.$$

which allows us to define the associated tensor operators, ${\hat{D}}_{m}$, according to

(103) $${\hat{d}}_{m}p^{\left(n\right)}\left(r\right)=\underset{\alpha \beta \gamma ..}{\sum^{}}{\left({\hat{D}}_{m}P^{\left(n\right)}\right)}_{\alpha \beta \gamma ..}R_{\alpha \beta \gamma ..}^{\left(n\right)}$$

where Equation (103) must define uniquely the ${\hat{D}}_{m}$, because the polynomial ${\hat{d}}_{m}p^{\left(n\right)}\left(r\right)$ is uniquely defined, and there is a one to one correspondence between polynomials and their equivalent tensors.

One iteration of Equation (103) gives

(104) $${\hat{d}}_{m^{\prime }}{\hat{d}}_{m}p^{\left(n\right)}\left(r\right)=\underset{\alpha \beta \gamma ..}{\sum^{}}{\left({\hat{D}}_{m^{\prime }}{\hat{D}}_{m}P^{\left(n\right)}\right)}_{\alpha \beta \gamma ..}R_{\alpha \beta \gamma ..}^{\left(n\right)}$$

Consider the case $m^{\prime }=m$, for which we have ${\hat{d}}_{m}={\hat{d}}_{m}{\hat{d}}_{m}$, given that ${\hat{d}}_{m}$ are projection operators. In this case, equating Equations (103) and (104) then implies that ${\hat{D}}_{m}{\hat{D}}_{m}={\hat{D}}_{m}$, and so ${\hat{D}}_{m}$ is also a projection operator.

Conversely, for $m^{\prime }\neq m$, we have that ${\hat{d}}_{m^{\prime }}{\hat{d}}_{m}=0$, because ${\hat{d}}_{m}$ are orthogonal projectional operators, in which case, Equation (104) must equal zero, which can only be possible for all $P^{\left(n\right)}$, $R^{\left(n\right)}$ if ${\hat{D}}_{m^{\prime }}{\hat{D}}_{m}=0$, again for $m^{\prime }\neq m$; from which we conclude that the ${\hat{D}}_{m}$ are *orthogonal* projection operators.

It can be seen from Equation (101) that the ${\hat{d}}_{0}$ operator projects polynomials into the space of harmonic polynomials of the same degree, with ${\hat{d}}_{0}h^{\left(n\right)}\left(r\right)=h^{\left(n\right)}\left(r\right)$ for any $h^{\left(n\right)}\left(r\right)$. Given that the tensor equivalent to a harmonic polynomial is traceless, the corresponding ${\hat{D}}_{0}$ operator must project tensors into the space of traceless tensors, i.e., we have that ${\hat{D}}_{0}=\hat{D}$, the detracing operator.

### 4.5. Conversion between the Tensor and Spherical Inner Products

This sub-section gives the conversion between the spherical inner product ${\left\langle p^{a\left(n\right)},p^{b\left(n\right)}\right\rangle }_{s}$, defined in Equation (53), and the tensor inner product, ${\left\langle P^{a\left(n\right)},P^{b\left(n\right)}\right\rangle }_{t}$, where $P^{a\left(n\right)}$ is the tensor equivalent to $p^{a\left(n\right)}\left(r\right)$, and similarly for $P^{b\left(n\right)}$ and $p^{b\left(n\right)}\left(r\right)$.

We begin by recalling from Equation (44) that any degree n homogeneous polynomial can be written as

(105) $$p^{\left(n\right)}\left(r\right)=\underset{\alpha \beta \gamma ..}{\sum^{}}P_{\alpha \beta \gamma ..}^{\left(n\right)}R_{\alpha \beta \gamma ..}^{\left(n\right)}$$

Substituting the above into the $\langle p^{a\left(n\right)},p^{b\left(n\right)}\rangle_{s}$ spherical inner product gives

(106) $$\begin{array}{c} \langle p^{a\left(n\right)},p^{b\left(n\right)}\rangle_{s}=\sum_{\alpha \beta \gamma ..}P_{\alpha \beta \gamma ..}^{a\left(n\right)}\sum_{\alpha^{\prime }\beta^{\prime }\gamma^{\prime }..}\langle R_{\alpha \beta \gamma ..}^{\left(n\right)},R_{\alpha^{\prime }\beta^{\prime }\gamma^{\prime }..}^{\left(n\right)}\rangle_{s}P_{\alpha^{\prime }\beta^{\prime }\gamma^{\prime }..}^{b\left(n\right)} \end{array},$$

which can be rewritten as

(107) $$\begin{array}{c} \langle p^{a\left(n\right)},p^{b\left(n\right)}\rangle_{s}=\langle P^{a\left(n\right)},\hat{K}P^{b\left(n\right)}\rangle_{t} \end{array}$$

where the self-adjoint tensor *operator* $\hat{K}$ is defined from its action on a degree *n* Cartesian tensor, $P^{\left(n\right)}$, according to

(108) $${\left(\hat{K}P^{\left(n\right)}\right)}_{\alpha \beta \gamma ..}=\underset{\alpha^{\prime }\beta^{\prime }}{\sum^{}}K_{\alpha \beta \gamma ..,\alpha^{\prime }\beta^{\prime }\gamma^{\prime }..}P_{\alpha^{\prime }\beta^{\prime }\gamma^{\prime }..},$$

where $\hat{K}$ has matrix elements

(109) $$K_{\alpha \beta \gamma ..,\alpha^{\prime }\beta^{\prime },\gamma^{\prime }..}=\langle R_{\alpha \beta \gamma ..}^{\left(n\right)},R_{\alpha^{\prime }\beta^{\prime }\gamma^{\prime }..}^{\left(n\right)}\rangle_{s}.$$

The components of $\hat{K}$ are easiest expressed in compressed notation, in which they are given by

(110) $$\begin{array}{c} K_{m,n}=\int^{}x^{m_{x}+n_{x}}y^{m_{y}+n_{y}}z^{m_{z}+n_{z}}dS\\ =4\pi \frac{\left(m_{x}+n_{x}-1\right)!!\left(m_{y}+n_{y}-1\right)!!\left(m_{z}+n_{z}-1\right)!!}{\left(2n+1\right)!!}e_{m+n} \end{array}$$

where $=m_{x}+m_{y}+m_{z}$ $=n_{x}+n_{y}+n_{z}$, and $e_{m+n}=1$ if $m_{x}+n_{x}$, $m_{y}+n_{y}$ and $m_{z}+n_{z}$ are all even, and $e_{m+n}=0$ otherwise, and where the integral above was solved using the methods for integrating polynomials over the unit sphere in ref. [25].

Although Equation (110) defines completely the matrix elements of $\hat{K}$, it is not in a very useful form. In the remainder of this section we will show how $\hat{K}$ can be put in a more useful form by writing it as a spectral sum in the projection operators, ${\hat{D}}_{m}$, defined in Section 4.5.

Let $\hat{R}=\hat{R}\left(\phi ,\theta ,\psi \right)$ be a rotation operator, parameterised in terms of Euler angles ϕ,θ,ψ say, which acts to rotate the system, where $\hat{R}$ acting on a polynomial rotates the polynomial by Euler angles ϕ,θ,ψ, i.e., $\hat{R}p\left(r\right)=p\left({\hat{R}}^{-1}r\right)$, where rotation of r can be achieved using orthogonal rotation matrices as described in Section 4.1. We also define the action of $\hat{R}$ on tensors, such that if $P^{\left(n\right)}$ is the tensor equivalent of the polynomial $p^{\left(n\right)}\left(r\right)$, then $\hat{R}P^{\left(n\right)}$ is the tensor equivalent of the polynomial $\hat{R}p^{\left(n\right)}\left(r\right)$.

We will now establish that both ${\hat{D}}_{m}$ and $\hat{R}$, and $\hat{K}$ and $\hat{R}$ always commute, i.e., $\left[{\hat{D}}_{m},\hat{R}\right]=0$ and $\left[\hat{K},\hat{R}\right]=0$. Furthermore, this will allow us to show that $\hat{K}$ must be a linear combination of ${\hat{D}}_{m}$.

We first show that $\left[{\hat{D}}_{m},\hat{R}\right]=0$. Recall from Section 4.4 that no rotation can move a polynomial, ${\hat{d}}_{m}p^{\left(n\right)}\left(r\right)$, in the *m*th subspace out of its subspace, which implies that ${\hat{d}}_{m}\hat{R}=\hat{R}{\hat{d}}_{m}$, or $\left[{\hat{d}}_{m},\hat{R}\right]=0$.

Now, given that any polynomial in the *m*th subspace (with associated projection operator ${\hat{d}}_{m}$) is equivalent to a tensor in its *m*th subspace (with associated projection operator ${\hat{D}}_{m}$), it also follows that no rotation of a tensor in its *m*th subspace can move that tensor out of the *m*th subspace. (If it could, we could transform a polynomial in the *m*th subspace into its corresponding tensor, rotate that tensor out of the *m*th subspace, and then transform back, which would mean that the *m*th subspace is not rotationally invariant.)

Thus, we also have that ${\hat{D}}_{m}\hat{R}=\hat{R}{\hat{D}}_{m}$, or $\left[{\hat{D}}_{m},\hat{R}\right]=0$, which was to be shown.

We will now show that $\left[\hat{K},\hat{R}\right]=0$. First note that because the spherical inner product is the integral of the product of two polynomials over the unit sphere, we have that rotating both polynomials by the same Euler angles must leave the inner product unchanged. We thus have that

(111) $$\langle p^{a}\left(r\right),p^{b}\left(r\right)\rangle_{s}=\langle \hat{R}p^{a}\left(r\right),\hat{R}p^{b}\left(r\right)\rangle_{s}.$$

From Equation (107), the LHS above can be rewritten as $\langle P^{a\left(n\right)},\hat{K}P^{b\left(n\right)}\rangle_{t}$. Further, we also have that the RHS above can be rewritten as

(112) $$\langle \hat{R}p^{a}\left(r\right),\hat{R}p^{b}\left(r\right)\rangle_{s}=\langle \hat{R}P^{a\left(n\right)},\hat{K}\hat{R}P^{b\left(n\right)}\rangle_{t}=\langle P^{a\left(n\right)},{\hat{R}}^{-1}\hat{K}\hat{R}P^{b\left(n\right)}\rangle_{t}$$

where the last part of Equation (112) above comes from applying the inverse rotation, ${\hat{R}}^{-1}$, to both sides of the inner product. We thus have that

(113) $$\langle P^{a\left(n\right)},\hat{K}P^{b\left(n\right)}\rangle_{t}=\langle P^{a\left(n\right)},{\hat{R}}^{-1}\hat{K}\hat{R}P^{b\left(n\right)}\rangle_{t}$$

which implies that $\hat{K}={\hat{R}}^{-1}\hat{K}\hat{R}$, or $\left[\hat{K},\hat{R}\right]=0$, which was to be shown.

We will now show that since $\left[\hat{K},\hat{R}\right]=0$ and $\left[{\hat{D}}_{m},\hat{R}\right]=0$, it follows that $\hat{K}$ must be a linear combination of ${\hat{D}}_{m}$.

Suppose that v is an eigenvector of $\hat{K}$ with eigenvalue α. Given that $\left[\hat{K},\hat{R}\right]=0$, we have that

(114) $$\hat{K}\hat{R}v=\hat{R}\hat{K}v=\alpha \hat{R}v$$

It follows that $\hat{R}v$ is also an eigenvector of $\hat{K}$, also with eigenvalue α. Thus, v must belong to a degenerate subspace of $\hat{K}$ spanned by all eigenvectors of $\hat{K}$ with eigenvalue α. Furthermore, this subspace is rotationally invariant, i.e., for any vector v in that subspace $\hat{R}v$ also belongs to that subspace. However, we have seen that the orthogonal irreducible rotationally invariant subspaces on the vector space of tensors are described by projection operators ${\hat{D}}_{m}$, so each one of the mutually orthogonal degenerate eigenspaces of $\hat{K}$ is one of the m spaces associated with the ${\hat{D}}_{m}$ projection operators.

Now, given that any symmetric matrix can be expressed as a linear sum: $A=\sum_{i}\lambda_{i}{\hat{P}}_{i}$, where ${\hat{P}}_{i}$ is the projection into the *i*th degenerate eigenspace, with associated eigenvalue $\lambda_{i}$, we have that $\hat{K}$ can be written in the form

(115) $$\hat{K}=\underset{m}{\sum^{}}\alpha_{m}{\hat{D}}_{m}$$

with the $\alpha_{m}$ to be determined.

As a check, we can take the commutator of both sides of Equation (115) above with respect to $\hat{R}$, which gives

(116) $$\left[\hat{K},\hat{R}]=\underset{m}{\sum^{}}\alpha_{m}[{\hat{D}}_{m},\hat{R}\right]=0$$

as expected.

Finally, substitution of Equation (116) above into Equation (107) gives

(117) $$\underset{m}{\sum^{}}\langle {\hat{d}}_{m}p^{a\left(n\right)},{\hat{d}}_{m}p^{b\left(n\right)}\rangle_{s}=\underset{m}{\sum^{}}\alpha_{n,m}\langle {\hat{D}}_{m}P^{a\left(n\right)},{\hat{D}}_{m}P^{b\left(n\right)}\rangle_{t}$$

where we have now included the index n in $\alpha_{n,m}$ given that it is possible these scalars also have a dependence on *n*, and where we have used

(118) $$\langle p^{a\left(n\right)},p^{b\left(n\right)}\rangle_{s}=\underset{m}{\sum^{}}\langle {\hat{d}}_{m}p^{a\left(n\right)},{\hat{d}}_{m}p^{b\left(n\right)}\rangle_{s}$$

However, Equation (117) must hold separately for each value of *m*, for the *m*th term on the LHS can only depend on the *m*th term on the RHS. So we have that

(119) $$\begin{array}{c} \langle {\hat{d}}_{m}p^{a\left(n\right)},{\hat{d}}_{m}p^{b\left(n\right)}\rangle_{s}=\alpha_{n,m}\langle {\hat{D}}_{m}P^{a\left(n\right)},{\hat{D}}_{m}P^{b\left(n\right)}\rangle_{t} \end{array}$$

which, apart from the $\alpha_{n,m}$ to be determined in the next sub-section (i.e., 4.6) for the *m* = 0 case, is our final form for the conversion between inner products.

### 4.6. Proportionality Constants for Conversion between the Tensor and Spherical Inner Products for the Case of Traceless Tensors and Harmonic Functions

In Section 4.5, we developed a general expression (Equation (119)) for the conversion between the spherical and tensor inner products. We will only need the *m* = 0 case for this present work, for which the ${\hat{D}}_{0}$ projection operator is the detracing operator, $\hat{D}={\hat{D}}_{0}$, and the associated ${\hat{d}}_{0}$ operator describes a projection onto the space of harmonic polynomials. Thus, for m = 0, Equation (119) can be written as

(120) $$\langle h^{a\left(n\right)},h^{\left(n\right)}\rangle_{s}=\alpha_{n,0}\langle H^{a\left(n\right)},H^{b\left(n\right)}\rangle_{t}$$

where $H^{\left(n\right)}$ are traceless tensors, and $h^{\left(n\right)}\left(r\right)$ are their associated harmonic polynomials.

In the present sub-section, it will be shown that the rank-dependent proportionality constants $\alpha_{n,0}$ are given by

(121) $$\alpha_{n,0}=\frac{4\pi n!}{\left(2n+1\right)!!}$$

Note that as the multipole interaction formulae are written in terms of traceless tensors, only the m = 0 case is required for this present work. However, the general approach described here should be extensible to results for higher orders.

Our ‘plan of attack’ is to fix its value by choosing the simplest rank n harmonic polynomials we can think of, which are arguably provided by the so-called *zonal spherical harmonics*, $r^{n}l_{n}\left(z/r\right)$, where $l_{n}\left(z\right)$ is the *n*th Legendre polynomial, and where $\Delta \left\{r^{n}l_{n}\left(z/r\right)\right\}=0$. We also have that the zonal spherical harmonics are symmetric about the z axis, which should make them particularly simple to work with.

Again, aiming for simplicity, we will set both $h^{a\left(n\right)}\left(r\right)$ and $h^{b\left(n\right)}\left(r\right)$ to the same zonal spherical harmonic.

(122) $$h^{a\left(n\right)}\left(r\right)=h^{b\left(n\right)}\left(r\right)=r^{n}l_{n}\left(z/r\right)$$

Now, using the properties of Legendre polynomials, we have that the LHS of Equation (120) is given by

(123) $$\langle l_{n},l_{n}\rangle_{s}={\int^{}}_{\phi =0}^{2\pi }{\int^{}}_{\theta =0}^{\pi }l_{n}^{2}\left(cos\left(\theta \right)\right)sin\left(\theta \right)d\theta d\phi =2\pi {\int^{}}_{z=-1}^{1}l_{n}^{2}\left(z\right)dz=\frac{4\pi }{2n+1}$$

where we have used the standard formula for Legendre polynomials that the integral of $l_{m}\left(x\right)l_{n}\left(x\right)$ over z=−1 to 1 is given by $2/\left(2n+1\right)\delta_{m,n}$.

We next turn to calculating the equivalent inner product on the RHS of Equation (120). Let $L^{\left(n\right)}$ be the zonal spherical harmonics in Cartesian tensor form, which using Equation (50) are given by $L^{\left(n\right)}=\left(1/n!\right)\nabla^{\left(n\right)}\left\{r^{n}l_{n}\left(z/r\right)\right\}$. Given the z symmetry of the zonal harmonics, we have that the $L^{\left(n\right)}$ must be proportional to $\hat{D}Z^{\left(n\right)}$, where $Z^{\left(n\right)}=\hat{z}\hat{z}\hat{z}..$. (No other choice would have the right symmetry.)

Taking the inner product of a rank n tensor, e.g., $A^{\left(n\right)}$, with $Z^{\left(n\right)}$ returns the $A_{zzz..}^{\left(n\right)}$ component, i.e., $\langle A^{\left(n\right)},Z^{\left(n\right)}\rangle_{t}=A_{zzz..}^{\left(n\right)}$, and we will now state two such inner products (to be proved at the end of this section), which will come in useful for the following.

(124) $$\langle L^{\left(n\right)},Z^{\left(n\right)}\rangle_{t}=1$$

(125) $$\langle \hat{D}Z^{\left(n\right)},Z^{\left(n\right)}\rangle_{t}=\frac{n!}{\left(2n-1\right)!!}$$

Using the last two identities above fixes the proportionality constant between $\hat{D}Z^{\left(n\right)}$ and $L^{\left(n\right)}$ to give

(126) $$L^{\left(n\right)}=\frac{\left(2n-1\right)!!}{n!}\hat{D}Z^{\left(n\right)}.$$

We can then write

(127) $$\begin{array}{l} \langle L^{\left(n\right)},L^{\left(n\right)}\rangle_{t}=\frac{\left(2n-1\right)!!}{n!}\langle L^{\left(n\right)},\hat{D}Z^{\left(n\right)}\rangle_{t}\\ =\frac{\left(2n-1\right)!!}{n!}\langle L^{\left(n\right)},Z^{\left(n\right)}\rangle_{t}=\frac{\left(2n-1\right)!!}{n!} \end{array}$$

where we have (i) used Equation (126) to exchange $L^{\left(n\right)}$ for $\hat{D}Z^{\left(n\right)}$, (ii) used the fact that $L^{\left(n\right)}$ is inside the traceless subspace to allow us to exchange $\hat{D}Z^{\left(n\right)}$ for $Z^{\left(n\right)}$, and (iii) used Equation (124) above.

Finally, substituting Equations (127) and (123) into Equation (120) fixes $\alpha_{n,0}=4\pi n!/\left(2n+1\right)!!$, which is Equation (121).

As promised, we will now provide the proofs of Equations (124) and (125).

To prove Equation (124), we use the identity

(128) $$\frac{\partial }{\partial z}\left\{r^{n}l_{n}\left(z/r\right)\right\}=nr^{n-1}l_{n-1}\left(z/r\right)$$

which can be proved by algebraic differentiation using the standard identity for the derivative of a Legendre polynomial: $l_{n}^{\prime }\left(x\right)=\left(n/\left(x^{2}-1\right)\right)\left(xl_{n}\left(x\right)-l_{n-1}\left(x\right)\right)$.

Thus, we have that

(129) $$\langle L^{\left(n\right)},Z^{\left(n\right)}\rangle_{t}=L_{zzz..}^{\left(n\right)}=\frac{1}{n!}\frac{\partial^{n}}{\partial z^{n}}\left\{r^{n}l_{n}\left(z/r\right)\right\}=1.$$

To prove Equation (125), we use the standard generating function for Legendre polynomials

(130) $$\frac{1}{{\left(1-2xt+t^{2}\right)}^{1/2}}=\overset{\infty }{\underset{n=0}{\sum^{}}}l_{n}\left(x\right)t^{n}$$

Substituting x=cos(θ) and t=r, where cos(θ)=z/r, and recognising that, under these substitutions, the LHS above is now equivalent to $|r-\hat{z}{|}^{-1}$, we have

(131) $$\frac{1}{\left|r-\hat{z}\right|}=\overset{\infty }{\underset{n=0}{\sum^{}}}l_{n}\left(cos\left(\theta \right)\right)r^{n}$$

However, the LHS above can also be Taylor-expanded as

(132) $$\frac{1}{\left|r-\hat{z}\right|}=\overset{\infty }{\underset{n=0}{\sum^{}}}\frac{{\left(-1\right)}^{n}}{n!}\langle Z^{\left(n\right))},\nabla^{\left(n\right)}\rangle_{t}\left\{\frac{1}{r}\right\}=\frac{\left(2n-1\right)!!}{n!r^{2n+1}}\overset{\infty }{\underset{n=0}{\sum^{}}}\langle Z^{\left(n\right))},R^{t\left(n\right)}\rangle_{t}$$

where we have made use of the expression for the Maxwell Cartesian spherical harmonics from Equation (11).

Comparing Equations (131) and (132), we deduce that

(133) $$\langle Z^{\left(n\right))},R^{t\left(n\right)}\rangle_{t}=\frac{n!r^{2n+1}}{\left(2n-1\right)!!}l_{n}\left(cos\left(\theta \right)\right)r^{n}$$

Now, setting r=1 and θ=0 means the LHS becomes equal to $\langle Z^{\left(n\right)},\hat{D}Z^{\left(n\right)}\rangle_{t}$, and RHS is given by n!/(2n−1)!!, which gives the required identity.

### 4.7. Ewald-Sum Terms

For completeness, the full Ewald sum is given by (following Smith [1]).

(134) $$\begin{array}{c} U=\frac{1}{2V}\sum_{k\neq 0}^{\infty }A\left(k\right){\left|\sum_{j}^{N}f_{j}\left(k\right)exp\left(-ik.r_{j}\right)\right|}^{2}+\\ \frac{1}{4\pi \epsilon_{0}}\sum_{l=1}\sum_{i=1}^{N}\sum_{j>i}^{\infty }G_{ji}^{l}\left(r^{ji}\right)B_{l}\left(r^{ji}\right)+\frac{1}{2}\sum_{i}^{N}\phi_{i}^{S} \end{array}$$

where the $G_{ji}^{l}\left(r^{ji}\right)$ terms are given by Equation (31) for Cartesians, and by Equation (69) for spherical harmonics, and where the kernel $B_{0}\left(r\right)=erfc\left(\epsilon r\right)/r$ is used, where ϵ is a width parameter, which controls the Gaussian width of the Ewald screening charges, and where the higher order B functions generated by Equation (25).

The k in Equation (134) are reciprocal-lattice vectors: $k=2\pi \left(n_{a}/L_{a},n_{b}/L_{b},n_{c}/L_{c}\right)$ for an orthogonal unit cell of dimensions $L_{a},L_{b},L_{c}$.

(135) $$f_{j}\left(k\right)=\underset{l=0}{\sum^{}}{\left(-i\right)}^{l}\langle M^{\left(l\right)},K^{\left(l\right)}\rangle_{t}$$

where $K^{\left(l\right)}$ is the tensor product $K^{\left(l\right)}=kkk..$

The $\phi_{i}^{S}$ in Equation (134) are so-called self terms, given by

(136) $$\phi_{i}^{S}=-\frac{1}{4\pi \epsilon_{0}}\underset{l=0}{\sum^{}}\frac{{\left(2\zeta^{2}\right)}^{l+1}}{\left(2l+1\right)\zeta \sqrt{\pi }}\langle M^{\left(l\right)},M^{\left(l\right)}\rangle_{t}$$

Note: A recent paper by Stamm et al. [26] provides a mathematically rigorous derivation of the self terms as given by Smith.

Both Equations (135) and (136) are in the form of inner products, and so can be readily expressed in either Cartesians or in spherical harmonics using Equation (61).

## 5. Conclusions

We have presented a non-technical—indeed, almost trivial—derivation of the multipole interaction in spherical harmonics, in a form suitable for use with Ewald-sum methods.

We began by summarising one derivation of the multipole interaction in Cartesians, most of which is not new to us, but goes back to the work of Smith and earlier, and continues up to Lin’s derivation of what we refer to as the multipole interaction generating formula.

We then introduced a diagrammatic method for visualising the multipole interactions, in where it was shown that the entire multipole interaction can be represented as a ‘sum over diagrams’, which arguably makes for a much more appealing representation of the interaction than thinking in terms of mathematical formulae alone.

Here, we admit that the results in this work could have been obtained without the aid of diagrams, but given that the diagrams show so clearly the structure of the interaction, we think they provide a valuable insight into what our various algebraic manipulations are actually doing.

Using what we believe to be a novel approach, the remainder of this work showed how the multipole interaction can be converted from Cartesians into spherical harmonics. This involved what was perhaps the key part of this paper, which was (i) the recognition that the multipole interaction in Cartesians involve expressions that can be written as inner products over tensors, (ii) showing that these tensor inner products are proportional to an inner product over the vector space of harmonic polynomials on the unit sphere, (iii) using this relation to convert the Cartesian tensor inner products to spherical inner products involving spherical harmonic polynomials.

The key expression here is Equation (54), which gives the proportionality relation between tensor and spherical inner products. Deriving this relation turns out to be a surprisingly non-trivial problem, but once it has been shown, it becomes a relatively straightforward task to convert the entire multipole interaction from Cartesians into spherical harmonics.

Our method relies on the use of existing tables of spherical harmonics to Cartesian transformations, which have already been given by Stone, and whose properties have been analysed extensively by Stone and others.

Another novelty of our method is that our ‘end-result’ formulae are not pure functions of the spherical harmonic multipoles, like seen in other approaches. Instead, our formulae are functions of split tensor components, which may have several indices, all of which are separately represented in spherical harmonics. However, what really distinguishes our approach from others is that our approach involves fully transforming *the between-tensor* contractions (bonds in the diagrams) into their spherical harmonic form, while respecting the underlying structure of the contractions; this structure which is particularly evident from the diagrammatic form.

It is surprising to us that such a straightforward conversion method appears to have been overlooked in the literature. It is not clear to us if this method will prove to give superior efficiency when compared to standard approaches, but it is faster than the Cartesian scheme, and it appears to us that our method is comparatively more straightforward and easier to implement than previous approaches. Furthermore, it is our hope that this work will prove a ‘less-painful’ route for the implementation of spherical-harmonic multipole expansions in computational-chemistry codes.

## Figures and Tables

**Table 1:** Spherical harmonics $q^{i\left(n\right)}\left(r\right)$ in Cartesians up to rank 3. Adapted from Stone, and normalised such that $∥Q^{i\left(n\right)}∥=1$, where $Q^{i\left(n\right)}$ are the tensor forms of the $q^{i\left(n\right)}\left(r\right)$ polynomials (see text for details). Here, we are using a simplified labelling scheme, in which the spherical harmonics, $q^{i\left(n\right)}$, are identified by their degree, (n), and an index, i, within each degree, where the index runs from 1..2n+1, and the ordering within each degree is (for our purposes) arbitrary.

Degree 0 $$q^{i\left(0\right)}=1$$
Degree 1 $$q^{1\left(1\right)}=x\;q^{2\left(1\right)}=y\;q^{3\left(1\right)}=z$$
Degree 2 $$q^{1\left(2\right)}=\left(\sqrt{6}/6\right)\left(3z^{2}-r^{2}\right)\;q^{2\left(2\right)}=\left(\sqrt{2}\right)xz\;q^{3\left(2\right)}=\left(\sqrt{2}\right)yz$$ $$q^{4\left(2\right)}=\left(\sqrt{2}/2\right)\left(x^{2}-y^{2}\right)\;q^{5\left(2\right)}=\left(\sqrt{2}\right)xy$$
Degree 3 $$q^{1\left(3\right)}=\left(\sqrt{10}/10\right)z\left(5z^{2}-3r^{2}\right)\;q^{2\left(3\right)}=\left(\sqrt{15}/10\right)x\left(5z^{2}-r^{2}\right)$$ $$q^{3\left(3\right)}=\left(\sqrt{15}/10\right)y\left(5z^{2}-r^{2}\right)\;q^{4\left(3\right)}=\sqrt{3/2}z\left(x^{2}-y^{2}\right)$$ $$q^{5\left(3\right)}=\sqrt{6}xyz\;q^{6\left(3\right)}=\left(1/2\right)x\left(x^{2}-3y^{2}\right)$$ $$q^{7\left(3\right)}=\left(1/2\right)y\left(3x^{2}-y^{2}\right)$$

**Table 2:** Cartesians in terms of spherical harmonics, up to rank 3. Adapted from Stone, such that the transformation is the inverse of Table 1.

Degree 2 $$x^{2}=-\left(\sqrt{6}/6\right)q^{1\left(2\right)}+\left(\sqrt{2}/2\right)q^{4\left(2\right)}y^{2}=-\left(\sqrt{6}/6\right)q^{1\left(2\right)}-\left(\sqrt{2}/2\right)q^{4\left(2\right)}$$ $$z^{2}=\left(\sqrt{6}/3\right)q^{1\left(2\right)}\;xy=\left(\sqrt{2}/2\right)q^{5\left(2\right)}$$ $$xz=\left(\sqrt{2}/2\right)q^{2\left(2\right)}\;yz=\left(\sqrt{2}/2\right)q^{3\left(2\right)}$$
Degree 3 $$x^{3}=\left(1/2\right)q^{6\left(3\right)}-\left(\sqrt{15}/10\right)q^{2\left(3\right)}\;x^{2}y=\left(1/2\right)q^{7\left(3\right)}-\left(\sqrt{15}/30\right)q^{3\left(3\right)}$$ $$xy^{2}=-\left(1/2\right)q^{6\left(3\right)}-\left(\sqrt{15}/30\right)q^{2\left(3\right)}\;y^{3}=-\left(1/2\right)q^{7\left(3\right)}-\left(\sqrt{15}/10\right)q^{3\left(3\right)}$$ $$\begin{array}{c} x^{2}z=\left(\sqrt{6}/6\right)q^{4\left(3\right)}-\left(\sqrt{10}/10\right)q^{1\left(3\right)}\;xyz=\left(\sqrt{6}/6\right)q^{5\left(3\right)}\\ y^{2}z=-\left(\sqrt{6}/6\right)q^{4\left(3\right)}-\left(\sqrt{10}/10\right)q^{1\left(3\right)}\;xz^{2}=2\left(\sqrt{15}/15\right)q^{2\left(3\right)}\\ yz^{2}=2\left(\sqrt{15}/15\right)q^{3\left(3\right)}\;z^{3}=\left(\sqrt{2}/5\right)q^{1\left(3\right)} \end{array}$$
